# “I am adhering to HIV treatment so that I can live to support her”: A qualitative study of upward intergenerational support in South Africa

**DOI:** 10.7189/jogh.14.04083

**Published:** 2024-05-10

**Authors:** Henning Schröder, Palesa Mataboge, Shannon A McMahon, F Xavier Gómez-Olivé, Enid J Schatz, Till Bärnighausen, Jan-Walter De Neve

**Affiliations:** 1 Heidelberg Institute of Global Health, Faculty of Medicine and University Hospital, University of Heidelberg, Heidelberg, Germany; 2 Medical Research Council/Wits Rural Public Health and Health Transitions Research Unit (Agincourt), School of Public Health, Faculty of Health Sciences, University of the Witwatersrand, Johannesburg, South Africa; 3 Department of International Health, Johns Hopkins Bloomberg School of Public Health, Johns Hopkins University, Baltimore, Maryland, USA; 4 Department of Public Health, College of Health Sciences, University of Missouri, Columbia, Missouri, USA; 5 Africa Health Research Institute, KwaZulu-Natal, South Africa; 6 Harvard Center for Population and Development Studies, Harvard University, Cambridge, Massachusetts, USA

## Abstract

**Background:**

Intergenerational family care, which was upended by the HIV epidemic in sub-Saharan Africa (SSA), may return to a pre-HIV era arrangement as access to antiretroviral therapy (ART) expands and treated adults can once again provide support for older household members. Empirical research has demonstrated positive ‘spillover effects’ of ART uptake from treated adults to younger generations, yet much less is known about the nature and breadth of such effects to older generations. This study explores the role and lived experiences among adults who take up ART and those of an older generation with whom they live.

**Methods:**

We conducted a qualitative study consisting of semi-structured interviews (n = 46) embedded in the Agincourt Health and Demographic Surveillance System (HDSS) in rural South Africa, between July and September 2022. We purposefully sampled two respondent categories: (i) young or middle-aged adults on ART (aged 18–59 years old); and (ii) older adults (aged ≥60 years old) who were affiliated with a young or middle-aged adult on ART. We used thematic content analysis to extract, code, and categorise relevant text by types of upward spillover effects from ART in younger adults to older adults. Quantitative data was extracted from the existing Agincourt HDSS database and matched to qualitative interview data based on Clinic link unique identifiers of study participants.

**Results:**

Mean age was 41 years among young or middle-aged adults (n = 29) and 72 years among older adults (n = 17). Among younger adults, time on ART ranged from five months to more than 21 years. Both young or middle-aged adults on ART and older adults reported positive spillover effects for older adults across five main tiers: caregiving, financial support, physical and mental health, living arrangements and household relationships, and stigma and reputation. Spillover challenges included financial costs and caregiving responsibilities following ART initiation of young or middle-aged adults, although these additional caregiving responsibilities were generally not perceived as particularly burdensome.

**Conclusions:**

ART is likely to benefit older adults in South Africa whose families are affected by HIV. This study identified a wide range of perceived spillover effects from ART in younger adults to older adults, including improvements to upward intergenerational support. These qualitative findings offer a guide to researchers, policymakers, and donors to capitalise on the broader societal effects of a large-scale health intervention to further support family structures and meet the needs of a growing older population.

The older population in sub-Saharan Africa (SSA) is expected to triple by 2050, representing the fastest growth rate globally of those aged 60 years and older [1]. In South Africa specifically, the older population is expected to more than double from 5 million in 2020 to over 12 million in 2050. While in high-income settings, an aging population can often rely on governmental bodies to provide financial, social and health care system support, in low- and middle-income countries (LMICs), older people often rely on family members, in particular their children, for care [2–4]. In SSA, intergenerational support is rooted in traditional patterns of care for older people. A manifestation of familial support entails co-residence, wherein older family members are cared for by younger generations living within the same home [5]. In SSA, about 75% of older people live with a young or middle-aged adult (18–59 years old) [6], and just about 9.7% of older people live alone (compared to 27.8% of lone, older dwellers in high income countries such as in Europe [7]). Global initiatives, including the United Nations Decade of Healthy Ageing (2021–2030), highlight the importance of intact family structures for the provision of old-age support. One strategy to meet the needs of an aging population will entail strengthening the skills and acumen of the younger generation in their ability to take care of older family members [8–10].

Prior to the availability of antiretroviral therapy (ART), the HIV epidemic caused a rapid decline of health and premature mortality, particularly among young and middle-aged adults [11]. The HIV epidemic hollowed out the middle generation in households leaving dependent household members without care [12–14]. AIDS-related deaths among adult children are associated with declining support for older people including future old-age support [15,16] and led to drastic changes in household composition [17], including the creation of ‘missing generation’ or ‘skipped-generation’ households where older adults live with orphaned grandchildren [18]. These changes led to a large, additional caregiving burden for older adults who not only lost their anticipated old-age support from young and middle-aged household or family members, but also had to take on additional roles to replace missing young and middle-aged adults who had provided financially and physically for the household or family [19–25].

The rapid roll-out of ART coverage in SSA has the potential to mitigate and even reverse the adverse effects of the HIV epidemic for the older population. As of 2022, 81% of people living with HIV received ART in SSA [26]. ART is shown to restore health and prevent AIDS-related mortality among treated individuals [27–30] and has been shown to have indirect effects on their household and family members, what we term ‘spillover effects’ [31]. Findings from Thailand, for example, suggest that ART among younger adults may lead to considerable benefits for older adults such as their parents or grandparents (also known as ‘upward’ spillover effects from ART among the younger generation to the older generation). These include fewer caregiving responsibilities and a reduction in older persons’ HIV-related expenditures for their adult children [32]. However, relatively little is known about such possible upward spillover effects from ART in younger adults to older adults in SSA. In a recent systematic scoping review on intergenerational spillover effects [31], we identified only one study which explicitly considered spillover effects from the younger to the older generation. The identified study was a multi-country quantitative study, which found that increased ART coverage was associated with improvements in the living arrangements of older adults, including fewer missing generation households [6]. The current study is therefore, to our knowledge, among the first to fill this gap in the literature and the first to explore perceptions of the role of ART for older people who are affected by ART among their family or household members in SSA.

## METHODS

### Study setting

This qualitative study was embedded within an existing Health and Demographic Surveillance System (HDSS) site, part of the MRC/Wits Rural Public Health and Health Transitions Research Unit (MRC/Wits-Agincourt Unit), located in Bushbuckridge, Mpumalanga Province in South Africa (Figures S1–S2 in the **Online Supplementary Document**). The Agincourt HDSS site encompasses a semi-arid rural area and is home to over 120 000 people living in 21 000 households and 31 villages as of 2023 [33]. The surveillance population has prospectively been followed for almost three decades including an annual census and a clinic-based databank called ‘Clinic link’. Clinic link captures health information of each patient visiting a clinic within the surveillance area and then links them to the census of the HDSS resulting in a robust research infrastructure. This structure facilitates community-based research on the roll-out of ART and intergenerational processes [34,35]. Embedding the study in the HDSS area allowed us to link data from our qualitative interviews with regularly collected socio-demographic data as well as clinical data on HIV outcomes.

The Primary Health Care system in the area includes a total of six clinics and a health center [35]. All of these health facilities have HIV counselling, testing and treatment services. Widespread ART access at public clinics began in early 2010s [36] and ART is now provided free of charge at all clinics, health centers, and district hospitals. To obtain ART, patients need to register at a clinic and then need to regularly pick up their treatment from that clinic in the future. In addition, the HDSS area is characterised by high levels of poverty and unemployment, leading to high rates of labor migration and reliance on remittances as an important source of income [33]. All individuals who are 60 years or older are eligible for a government social grant referred to as the ‘Older Person’s Grant’ (also called old-age pension grant), which provides up to about 2100 South African rand per month (about 110 US dollars) [37]. The South African Social Security Agency awards Older Person’s Grants based on a means test. Additional details on the Older Person’s Grant and means test are available in Text S1 in the **Online Supplementary Document**.

### Study design

Our overarching aim was to gain a better understanding of potential upward spillover effects from ART in young or middle-aged adults to older adults. Our specific research question was ‘How does ART among young or middle-aged adults living with HIV affect their older household and family members (whether the older adult is also living with HIV or not) in rural South Africa?’. To address this question, we conducted an exploratory qualitative study to elicit the perceptions of young or middle-aged adults on ART and their older household members. We conducted one-on-one semi-structured interviews between July and September 2022 to gain an in-depth understanding of the respondents’ feelings and beliefs towards the research topic. Given the sensitivity of the topic, one-on-one in-depth interviews offered the advantage of obtaining great depth of understanding in the topic of interest in a private environment.

### Study population

We purposively sampled two respondent categories to elicit the perceptions of multiple generations. These two categories included: (i) young or middle-aged adults (aged 18–59 years old) living with HIV and treated with ART who have an older household or family member (aged 60 years and older); and (ii) older adults (aged 60 years and older) who have a young or middle-aged household or family member living with HIV and who is treated with ART [38]. Young or middle-aged adults and older adults were paired in ‘dyads’ using a required existing relationship through co-residence within the same household or kinship (eg, adult child and their older parent). Our sample was made up of respondents who were living within the Agincourt HDSS area, allowing us to obtain demographic and clinical data on HIV status and treatment outcomes using the existing HDSS Clinic link data (**Table 1**, Table S1 in the **Online Supplementary Document**). Not all study participants belonged to a pair because of (i) the exclusion of respondents based on one of the study criteria listed above; (ii) missing consent of young or middle-aged respondent to interview the older adult; and (iii) the unavailability of the potential respondent during the study period. Respondents in each category were purposively sampled by age and gender. Our sampling strategy used non-probability sampling and is therefore not representative of a broader population. The HIV status of older adults and the duration on ART among younger adults on ART were not inclusion criteria in this study.

**Table 1:** Selected socio-demographic characteristics of study participants*

Subsample†	Young or middle-aged respondents‡	Old-age respondents§	Pooled sample
	**(n = 29)**	**(n = 17)**	**(n = 46)**
Age in years, mean (range)	41 (18–57)	72 (60–85)	53 (18–85)
Gender			
*Female*	14 (48%)	13 (76%)	27 (59%)
*Male*	15 (52%)	4 (24%)	19 (41%)
Educational attainment			
*None*	1 (3%)	3 (18%)	4 (9%)
*Primary*	7 (24%)	4 (24%)	11 (24%)
*Secondary*	11 (38%)	0 (0%)	11 (24%)
*Unknown*	10 (34%)	10 (59%)	20 (43%)
Marital status			
*Single*	14 (48%)	0 (0%)	14 (30%)
*Married*	8 (28%)	2 (12%)	10 (22%)
*Divorced*	5 (17%)	0 (0%)	5 (11%)
*Widowed*	2 (7%)	15 (88%)	17 (37%)
Household size			
*Number of household members, mean (range)*	5.2 (1–12)	4.4 (1–14)	4.9 (1–14)
*Unknown*¶	2 (7%)	5 (29%)	7 (15%)
Co-residence between generationsǁ			
*Yes*	19 (66%)	12 (71%)	31 (67%)
*No, same community*	9 (31%)	5 (29%)	14 (30%)
*No, different community*	1 (3%)	0 (0%)	1 (2%)
Social security programmes			
*Child Support Grant*	8 (28%)	0 (0%)	8 (17%)
*Older Person’s Grant*	0 (0%)	15 (88%)	15 (33%)
*Social Relief of Distress grant*	2 (7%)	1 (6%)	3 (7%)
*Unemployment Insurance Fund*	1 (3%)	0 (0%)	1 (2%)
*Road Accident Fund*	1 (3%)	0 (0%)	1 (2%)
*None received*	15 (52%)	1 (6%)	16 (35%)
*Unknown*	2 (7%)	0 (0%)	2 (4%)
Primary occupation			
*Domestic chores*	16 (55%)	11 (65%)	27 (59%)
*Agricultural and forestry labour*	2 (7%)	0 (0%)	2 (4%)
*Traditional healer (‘sangoma’)*	1 (3%)	0 (0%)	1 (2%)
*Construction, manufacturing, and transport*	6 (21%)	0 (0%)	6 (13%)
*Scholar*	1 (3%)	0 (0%)	1 (2%)
*Other*	1 (3%)	2 (12%)	3 (7%)
*None*	2 (7%)	4 (24%)	6 (13%)

*See Table S1 in the **Online Supplementary Document** for selected HIV-related characteristics of all respondents.

†Percentages might not add up to 100 due to rounding.

‡Young or middle-aged was defined as ages 18–59 y old.

§Old-age was defined as ages 60 y or older.

¶The Agincourt HDSS database and interview transcripts yielded no data regarding number of household members for these participants.

ǁCo-residence was defined as between a young or middle-aged adult and an older adult.

### Recruitment

First, we recruited participants at selected clinics located in the HDSS area (Figure S2 in the **Online Supplementary Document**). Clinics were chosen based on the volume of total patients on ART in a given year and accessibility to the study team. With the help of clinic staff, we identified HIV patients of all adult ages (≥18 years old). Second, patients who were found to be eligible and who consented to participate in the study were asked to contact household or family members for permission to be approached by the study team at their houses leading to recruitment of respondents at the household. Our aim was to recruit pairs (dyads) of participants with one young or middle-aged respondent and one older respondent in each household or family. In total, we recruited 56 eligible respondents including 32 young or middle-aged adults and 24 older adults. We further excluded five older adults who, despite the consent of the young or middle-aged adult, were not informed about the HIV status of their younger household or family member and two older respondents who withdrew their consent during the interview. We also excluded two younger respondents who were found to be mentally ill and one younger respondent who was found to be under the influence of alcohol during the interview. Our final sample for the analysis consisted of 46 respondents (29 young or middle-aged adults and 17 older adults) including 15 pairs of a younger adult and affiliated older adult. We sought at least about 45 interviews to be sufficient to inform the research question [39]. Recruitment and interviews were led by two locally trained female fieldworkers of the MRC/Wits-Agincourt Research Unit with experience in qualitative research and who live in the HDSS area. We conducted a three-day training for fieldworkers, including important aspects of the study context and training in recruitment and qualitative data collection methods.

### Data sources

#### Qualitative data

Prior to the interviews, we obtained written informed consent from all participants. Interviews were conducted either at a private place at the clinic or at home based on respondents’ preference. All participants were assigned with a study code to ensure confidentiality. Fieldworkers conducted one-to-one in-depth interviews in the local language Xitsonga, which typically lasted between 30 and 60 minutes. Semi-structured interview guides were designed in Xitsonga, separately for younger and older adults (Files S1–S2 in the **Online Supplementary Document**). Interview guides covered respondents’ living arrangements, their experiences with HIV and AIDS, and how ART affected their living conditions. Respondents were prompted with open-ended questions and encouraged to provide elaborate responses. As a methodological innovation, we designed a ‘Timeline of Support’ prior to the study to serve as a visual aid to respondents during the interviews (Files S3–S4 in the **Online Supplementary Document**). Both younger and older respondents were instructed to indicate on this timeline the intensity of support from younger adults to older household or family members provided at different times of the HIV and treatment status of younger adults (pre-HIV diagnosis, HIV diagnosis, start of ART and present). Throughout data collection, the interview guide was updated based on new information gained from the fieldworkers during briefings and debriefings before and after each interview. Systematic debriefings entailed goal-oriented discussion of data immediately after it was collected and allowed us to gain immediate insights, correct course, and disseminate emerging data more quickly within the study team, other researchers, and the local community [40]. Interviews were audio-recorded, transcribed, and translated into English.

#### Quantitative data

The data team of the MRC/Wits-Agincourt Research Unit extracted relevant socio-demographic and clinical information on study participants from the existing Agincourt HDSS database [35]. We extracted data on age, gender, marital status, education, primary occupation, household composition, co-residence, receipt of any governmental social grants (such as the Older Person’s Grant and Social Relief of Distress Grant), the presence of HIV-related symptoms and, if applicable, date of HIV diagnosis and start date of HIV treatment. Quantitative data were matched and harmonised with qualitative interview databased on Clinic link unique identifiers using a Research Electronic Data Capture (REDCap) study database [41].

### Analysis

Transcripts of interviews were analysed using an inductive thematic analysis approach [42–44]. We defined thematic analysis as a ‘method for identifying, analysing and reporting patterns (themes) within data’ [44]. Thematic analysis aims to search for and identify common threads that extend across interviews. We decided on thematic analysis over other potential methods as it offered considerable flexibility in analysing interview data and determining themes. Transcripts were read several times to familiarise with the data. Relevant text related to the research question was extracted, coded, and thematically grouped into identified themes. We verified whether the themes worked in relation to the coded extracts and entire data set. As overarching themes, we considered different pathways of how ART among young or middle-aged adults may affect older adults. Spillover effects were defined broadly as any change that ART among young or middle-aged individuals induces to the health, development, socioeconomic situation, well-being or living arrangements of older household or family members [31]. We then summarised our qualitative findings by the types of spillover effect which were identified based on our analysis of transcripts. In the writing of this manuscript, we closely linked identified summaries of themes with quotes from participants to guarantee proximity of what was reported by participants during the interviews and the identified themes (‘to stay close to what respondents say’). We considered both positive and negative consequences of ART and changes over time. We sought to triangulate the perspectives of two generations to provide a more comprehensive picture of possible upward spillover effects of ART. We generally refrained from disaggregating our results across additional socio-demographic dimensions (such as the respondent’s gender or primary occupation) since these qualitative results were unlikely to reach saturation when using smaller subsamples [39]. Qualitative data was analysed by the first author under the guidance of all co-authors using QSR software NVivo 13.

### Ethical clearance

We obtained ethical approval of the Institutional Review Boards of the University of Heidelberg in Germany (S-321/2018), the Human Research and Ethics Committee (Medical) from the University of the Witwatersrand, Johannesburg (M190461) and the Human Research and Ethics Committee from the Mpumalanga Province in South Africa (MP-201908-005).

## RESULTS

### Descriptive results

In **Table 1**, we report selected socio-demographic characteristics of all study respondents. Among the 46 respondents included in the analysis, the mean age among young or middle-aged adults was 41 years (range: 18–57 years) and 72 years among older adults (range: 60–85 years). In the pooled sample, 27 respondents were female (59%) and 19 were male (41%). In most cases, younger adults were the adult child (66%) to the index older adult, followed by grandchild (14%), spouse (10%), daughter-in-law (7%) or niece (3%). Most young or middle-aged respondents (66%) shared the same household with the index older adult, 31% lived in the same village and only one younger respondent (3%) had to travel to the neighbouring village to visit old-aged parents. 93% of the younger or middle-aged respondents had an occupation compared to 76% of the older age group. Except for two older women (>80 years old), all female respondents were still engaged in domestic chores and further activities such as the collection of water and firewood as well as farming. Additionally, one respondent was working as a traditional healer, one respondent was cooking meals in a school, and other respondents had small businesses (such as selling weaved mattresses or nuts). Only one male respondent had a working contract. Other male respondents were involved in domestic activities, self-employed, or pursued occasional jobs such as construction work, herding cattle, or car washing. As a result of a lack of regular financial income, many households depended on social security programs such as the Older Person’s Grant (88%) or Child Support Grant (28%).

In addition, in Table S1 in the **Online Supplementary Document**, we report selected HIV-related characteristics. Among the total of 46 respondents, all 29 young or middle-aged respondents were living with HIV. In the older respondent group of 17 participants, 3 respondents were living with HIV and on ART themselves. Most younger respondents had tested for HIV because of sickness (48%). All younger respondents were treated with ART, ranging from five months on ART to more than 21 years on ART. Two of the younger respondents had been on ART since early childhood. All respondents on ART reported to adhere to HIV treatment implying that they took medication regularly and did not miss any clinic appointments at the time of the interview. In addition to information on HIV testing, diagnosis, and treatment duration from clinics, we extracted relevant information about disclosure of HIV status to other individuals from our interview transcripts (Table S1 in the **Online Supplementary Document**). Although we did not explicitly ask about all individuals to whom HIV status was disclosed, we report whether HIV status was disclosed to at least one household or family member. All respondents in our study sample had disclosed their HIV status to at least one family member except for one younger respondent.

### Young or middle-aged adults on ART and support for older adults over time

Intergenerational support from younger to older generations was considered a fundamental aspect of coexistence among household and family members, rooted in cultural expectations among both younger and older respondents.

Interviewer (I): *Who will be taking care of you in the future?*

Participant (P): *(Name of son), because he is the youngest and as tradition demands that his brothers should move out to have their own households then the youngest should remain behind to take care of the family house.* – Female older respondent (60 y)

Several older respondents emphasised the importance of receiving support from their young or middle-aged family members, such as this older woman:

P: *… my daughter (name of daughter) is here for me. She even built a big bedroom for me here, so I am not worried about it. She is the one who took me in after she has seen that I am getting very old, and I am unable to do things for myself then she stepped up for me.* – Female older respondent (85 y) with daughter (47 y)

Respondents described how these patterns of intergenerational support were threatened by an HIV diagnosis among younger adults. Among older respondents, some had experienced declining support from younger adults because of health declines induced by HIV. Reports of young or middle-aged children dying due to AIDS also emerged.

I: *How much support did you receive from (name of son) at the time of his HIV diagnosis?*

P: *He was not supporting me.*

I: *What could be the reason?*

P: *It was because he was sick and not working and I was the one who was supporting him during those times.* – Female older respondent (77 y) with son (50 y)

The initiation and consistent ART uptake among younger adults represented the premise to return to or remain in good health and thus sustain the living together between younger and older adults. In turn, good health allowed young or middle-aged respondents on ART to continue to support their older family members in various ways and to do the things that are expected within their community, regardless of their HIV infection.

P: *What I have realized about this HIV infection is that when you adhere to HIV treatment you will be able to do everything you want to do and if you don’t adhere to it, you won’t make it to life.* – Male middle-aged respondent (50 y) with mother (77 y)

P: *I see good things from my son ever since he started ART, he is supporting me he does not give me problems and his well-being is very good.* – Female older respondent (68 y) with son (37 y)

The perceived impact of ART to guarantee support for older adults differed among study respondents and depended on whether respondents had experienced disrupted intergenerational support structures due to HIV-related symptoms among younger family members. Among the 29 young and middle-aged respondents, 22 (76%) reported to have never or only mildly experienced HIV-related symptoms (Table S1 in the **Online Supplementary Document**). An early HIV diagnosis and initiation on ART allowed these younger adults to remain in good health without major changes to their living conditions. As a result, these younger adults explained that neither HIV nor ART influenced the level of support they provided to older household or family members.

I: *How has this (HIV) treatment affected your ability to support them (parents)?*

P: *The way I used to live, I still live that way and when they call me to come to them, as long as I am not doing anything, I rush to them to do whatever they ask me to do.* – Female young adult (31 y) with father (63 y)

Similarly, older respondents did not attribute changes in intergenerational support structures to the HIV treatment of their related young or middle-aged adults.

P: *I was receiving full support and was happy that my daughter in-law will have a life due to the HIV treatment. Being diagnosed at an early stage saves you from lot of things. There were no changes in her, she is still the same person who she was from the time she joined us to live with (before her HIV diagnosis).* – Female older respondent (60 y) with daughter-in-law (35 y)

This was also reflected in the responses of participants to the ‘Timeline of Support’ interview tool. Respondents indicated few noticeable changes to the intergenerational support provided between the time prior to HIV diagnosis, treatment start, and until the present. In contrast, however, younger adults who had fallen severely sick due to their HIV-infection (24% of young or middle-aged respondents in our study sample), experienced not only a decrease in their ability to support older family members but on the contrary were highly dependent on support from other family members (mostly from older parents such as their mothers).

I: *How much did you support your mother at the time of your HIV diagnosis?*

*P: I was unable to support her; I couldn’t even give her a single support because I was very sick. Instead, she was the one who was giving me support.* – Male middle-aged respondent (50 y) with mother (85 y)

Other respondents also indicated a larger amount of support provided to older adults prior to HIV diagnosis in younger adults. This was followed by a reduction in support during the time of sickness and initiation of treatment followed by an increase in support in the present. Respondents described how the duration of HIV treatment initiation and, more so, recovery determined the amount of support young or middle-aged individuals were able to provide to their older household and family members. For example, a middle-aged respondent who initiated ART only months ago, had not yet fully recovered from recent HIV-related illness, and was unable to return to work and provide support for older family members.

I: *How much support are you giving to your parents today?*

P: *I cannot support them now because I am sick, but they are the ones that are supporting me.*

I: *How much did you support you parents in the past before your HIV diagnosis?*

P: *There is so much that I was providing for them because I was not sick at those times, I gave them full support.*

I: *How much did you support your parents at the time of your HIV diagnosis?*

P: *I was unable to support them, I was sick and not working.*

I: *How much did you support your parents at the time you started taking HIV treatment?*

P: *Still I am unable to support them. I am still not that well and I have not yet gone back to work because my legs are in pains and being a car mechanic demands you to be standing most of the time.* – Male middle-aged respondent (40 y) with father (68 y)

In the long run, however, intergenerational support structures returned to the pre-HIV period. A younger adult who had been on ART for many years following a period of severe sickness, reported supporting his older mother the way he used to prior to HIV infection.

I: *How much support are you giving to your mother today?*

P: *Ever since I have been better, I am giving her full support I am doing everything possible to maintain her needs.* – Male middle-aged respondent (50 y) with mother (85 y)

Respondents also reported on the role of ART in future old-age support, which was seen as a kind of life saver that ensured sustained support for their older family members.

P: *I am adhering to HIV treatment so that I can live to support her (mother) till she dies, she needs me more than anything that’s why I stick to ART.* – Female middle-aged respondent (57 y) with mother (84 y)

### Types of spillover effects from ART in younger adults to older adults

**Figure 1:**
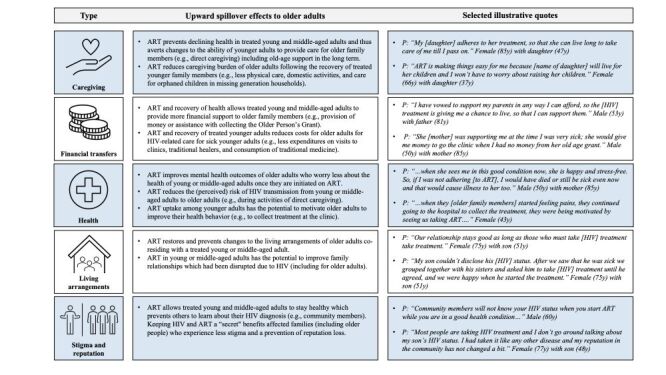
Types of upward spillover effects from young or middle-aged adults (18–59 years old) on antiretroviral therapy to older adults (60 years and older), reported by respondents (n = 46). The left column summarises key findings for each type of spillover effect supported by exemplary quotes in the right column (*P* = Participant). Additional descriptions and supportive quotes are provided in Text S2 in the **Online Supplementary Document**.

Both younger and older adults reported spillover effects across five main tiers: caregiving, financial transfers, health outcomes, living arrangements, and stigma and reputation (**Figure 1**, Text S2 in the **Online Supplementary Document**). Respondents generally reported positive effects of ART for older household and family members. Improved health conditions, for example, allowed younger adults to generally provide more caregiving and financial support to older adults in contrast to the time of limited health prior to ART initiation. HIV-related caregiving tasks for older adults (such as for orphaned children in missing generation households) were also reduced once younger adults initiated ART and recovered from HIV-related symptoms. In addition, improved health conditions allowed older people to worry less about the health of their treated adult relatives living with HIV. Moreover, ART was reported to prevent detrimental changes to family relationships and the reputation of older adults.

Besides the beneficial spillover effects, respondents also noted potential negative consequences for older adults. Those potential detrimental effects were mostly related to caregiving and financial transfers. Older adults, for example, took great effort in supporting ART initiation and adherence among younger adults, provided direct care in case of a gradual recovery from HIV-related symptoms, as well as financial and nutritional support for younger adults who initiated ART. Starting treatment did not entirely remove caregiving tasks by older adults for younger adults but rather led to a shift from taking care of HIV-related symptoms to supporting the treatment and adherence of younger adults on ART, either directly (through reminding their adult children to take treatment) or indirectly (through buying groceries and preparing meals for their adult children). There were also financial costs to support transport to the HIV treatment clinic or to support the uptake of ART with nutritious food. Nevertheless, despite the financial and opportunity costs associated with ART, older respondents generally did not perceive these efforts as a burden in view of the live-saving benefits of ART.

## DISCUSSION

Based on in-depth semi-structured interviews with young or middle-aged (18–59 years old) and older adults (60 years and older) residing in the Agincourt HDSS area in rural South Africa, we explored how ART among younger adults affected the well-being of older household and family members. While all younger adults were on ART (inclusion criteria), we elicited the perspectives of older adults regardless of their HIV status or whether they themselves were on treatment or not. This approach complements the traditional focus of ‘modern medicine’ which emphasises the effects of the treatment on the treated individual but misses potential ripple effects within the broader community (which we termed spillover effects) [31]. Employing a thematic analysis approach, we find that both respondent groups reported considerable and generally positive spillover effects of ART to older adults including effects on older adult’s support structures over time, caregiving and financial support, health and well-being, living arrangements and relationships, and reputation in the community. Older adults considered that they largely benefited from the HIV treatment of younger adults in the household.

Consistent uptake of ART among the younger generation had the potential to further mitigate and help reverse the burden of the HIV-epidemic for older adults [32]. Indeed, the decline in intergenerational support for older people, which had been limited by HIV-related sickness among young or middle-aged individuals [15], was restored following the recovery of younger adults initiated on ART. Antiretroviral therapy allowed younger adults to care for older adults regardless of their HIV infection. Moreover, ART uptake and recovery among the younger generation meant a reduction in parental caregiving as older adults were relieved from an additional caregiving burden to sick younger adults living with HIV [45–47] and for orphaned children living in missing generation households (such as their grandchildren) [6,17,32,48]. Furthermore, ART meant a great relief. Older adults had endured much suffering caused by the HIV epidemic. Many had experienced AIDS-related deaths within the community or among their own family [12,14,49]. Antiretroviral therapy allowed older adults to worry less about the health of their treated young and middle-aged adults in their household and family as well as to experience less stigma by community members [32]. Older adults were also potentially more motivated to seek health care and adhere to their own treatment seeing their younger family members adhering to ART [50]. Antiretroviral therapy also had the potential to restore disrupted living arrangements and relationships within the family and household [51,52]. Lastly, the widespread availability of ART, including among their young and middle-aged family members, known to effectively control HIV, led to a perception change of older adults with respect to the mortality risk evaluation of HIV and AIDS [53].

Following major policy changes, such as the ART same-day initiation in 2017 in South Africa [54], the number of people living with HIV that experience HIV-related symptoms has declined [55]. In this study, most younger adults received their HIV diagnosis and HIV treatment prior to the onset of severe sickness. Moreover, many of them had never (45%) or only mildly (31%) experienced HIV-related symptoms. As a result, these younger adults did not report major changes to their health following ART and did not perceive major consequences of ART for their living arrangements or their ability to provide support for older family members, apart from the effort required for treatment adherence. For these respondents, the primary benefit of ART was that life could continue ‘as usual’ without dramatic interruptions and any perceived changes would only be noticeable in the absence of or non-adherence to ART. These younger adults on ART were normally more concerned about other issues than their HIV diagnoses, including other diseases (such as diabetes and hypertension), as well as the broader socio-economic conditions of the region (such as high unemployment and poverty) [53].

To our knowledge, only two other studies explicitly investigated upward spillover effects of ART, including in SSA [6] and Thailand [32]. The study from SSA, for example, involved nearly 300 000 older individuals (aged 60 years or older) across several countries and assessed the relationship between country-level ART coverage and changes in co-residence between working-age and older adults in SSA. The study found that the expansion of ART coverage in SSA was associated with a significant decrease in the number of older adults living alone without a younger or middle-aged adult in the same household [6]. An increase in ART coverage was also associated with more working-age adults in households with at least one older person and a reduction in the number of missing generation households. Our qualitative results may help explain some of these quantitative findings in the context of South Africa. The respondents in our study acknowledged the life-saving impact of ART, particularly in light of AIDS-related deaths experienced by respondents in their social environment. ART was perceived to influence the living arrangements of older adults, with various downstream implications.

### Study limitations

This study has some limitations. First, our approach of taking the younger adult as the index case (the treatment recipient) and exploring spillover effects on older adults (the recipient of spillover effects) assumes that changes to the health of younger adults may have consequences for older adults. However, the assumption that young or middle-aged individuals take the central role within families as the primary provider may not be applicable to all households. The depiction of people over the age of 60 years to be ‘dependent’ on support may not hold true in all cases. Second, the study setting has some distinct characteristics compared to other settings in SSA, such as the public welfare system and health care infrastructure. The availability of the South African Old Person's Grant may alter intergenerational support structures. Additionally, the coverage of HIV treatment clinics in South Africa including in the study region might be higher compared to other rural areas in SSA where treatment adherence may be more challenging. South Africa is among the wealthiest countries in Africa and may be able to invest more in old-age pensions and health care infrastructure than other countries in SSA. The findings of this study may therefore not be generalisable to other demographic or socio-economic contexts. Third, support structures of older people are fundamentally changing in SSA [3,56]. Urbanisation, for example, resulted in many older people living alone, including in rural areas. A transformation of social values has changed the traditional pattern of younger adults to care for older household members [56]. Since our study purposively sampled younger and older adults who were living in the Agincourt HDSS area, we did not map effects of ART on intergenerational support structures in the context of family members who do not live closely to each other. Fourth, qualitative interviews may be particularly susceptible to social desirability bias [57]. Younger generations may be inclined to exaggerate the amount of support they provide to older family members in accordance with traditional expectations. In our qualitative interviews, for example, a working-age respondent claimed to provide significant support to his older mother, while the mother reported receiving no support and only seeing her son when he came for food or to spend time. Interviewing participant dyads (eg, mother and son), however, had the advantage of obtaining two perspectives on the same relationship making it possible to contextualise respondents’ answers. We also conducted regular debriefing sessions [40] after each interview which provided additional opportunities to detect and further mitigate potential social desirability bias.

### Implications for future research

A better understanding of the broader societal effects of health interventions is needed to capitalise on the positive spillover effects while mitigating potential detrimental spillover effects [31]. Building on the qualitative findings of this study, future research could, for example, quantify the level and extent of upward spillover effects of ART for older adults. These estimates can also inform cost-benefit analysis for HIV treatment programmes. Since global initiatives are scaling down on investments in ART [58], additional empirical evidence on the benefits of ART in treated individuals for household and family members can further justify ongoing investments in HIV treatment programs in the region. Additionally, our findings can serve as a guide for future research on other large-scale health interventions (beyond ART). The reduction in morbidity and mortality and a shift in disease perception as two main sources of spillover effects to older adults, which we identified in our study, may apply to other health interventions and for communicable and non-communicable diseases alike.

## CONCLUSIONS

In SSA, the world’s largest HIV treatment initiative is yielding considerable advantages, benefiting not only those individuals receiving treatment but also their household and family members. The uptake of ART may, in part, reverse the adverse societal effects of the HIV epidemic. These ripple effects of ART for household and family members are substantial and warrant further exploration, particularly among older generations, an area of research which has been largely underexplored so far. In this rural community in South Africa, the uptake of ART resulted in a wide range of perceived spillover effects for older adults, including improvements in caregiving and financial support for older adults, their health outcomes, living arrangements, household relationships, as well as improvements in the reputation of older adults in the local community. Considering the growing aging population in SSA, a better understanding of the broader societal impact of ART appears crucial in addressing the living conditions and well-being of the older generations.

## Additional material


Online Supplementary Document

## References

[1] He W, Aboderin I, Adjaye-Gbewonyo D. Africa Aging: 2020. US Census Bureau, International Population Reports, P95/20-1, US Government Printing Office, Washington, DC. 2020.

[2] Bongaarts J Zimmer Z Living arrangements of older adults in the developing world: an analysis of demographic and health survey household surveys. J Gerontol B Psychol Sci Soc Sci. 2002;57:S145–57. 10.1093/geronb/57.3.S145 11983741

[3] Aboderin I Decline in material family support for older people in urban Ghana, Africa: understanding processes and causes of change. J Gerontol B Psychol Sci Soc Sci. 2004;59:S128–37. 10.1093/geronb/59.3.S128 15118018

[4] Kohler IV Kohler HP Anglewicz P Behrman JR Intergenerational transfers in the era of HIV/AIDS: Evidence from rural Malawi. Demogr Res. 2012;27:775–834. 10.4054/DemRes.2012.27.27 23606809 PMC3628805

[5] Curreri NA McCabe L Robertson J Aboderin I Pot AM Keating N Family beliefs about care for older people in Central, East, Southern and West Africa and Latin America. International Journal of Care and Caring. 2022;1:1–21.

[6] De Neve JW Karlsson O Coetzee L Schröder H Subramanian SV Bärnighausen T Antiretroviral therapy coverage associated with increased co-residence between older and working-age adults in Africa. AIDS. 2018;32:2051–7. 10.1097/QAD.0000000000001917 29894389 PMC7293712

[7] United Nations, Department of Economic and Social Affairs, Population Division. Living arrangements of older persons: a report on an expanded international dataset (ST/ESA/SER.A/407). 2017.

[8] World Health Organisation. UN Decade of Healthy Ageing. 2023. Available: https://www.who.int/initiatives/decade-of-healthy-ageing. Accessed: 1 March 2024.

[9] African Union. Draft Policy Framework And Plan Of Action On Ageing. 2022. Available: https://au.int/sites/default/files/newsevents/workingdocuments/41106-wd-DRAFT_POLICY_FRAMEWORK_AND_PLAN_OF_ACTION_ON_AGEING-_ENGLISH_0.pdf. Accessed: 1 March 2024.

[10] Aboderin I Hoffman J Families, Intergenerational Bonds, and Aging in Sub-Saharan Africa. Can J Aging. 2015;34:282–9. 10.1017/S0714980815000239 26300188

[11] Piot P Bartos M Ghys PD Walker N Schwartländer B The global impact of HIV/AIDS. Nature. 2001;410:968–73. 10.1038/35073639 11309626

[12] Knodel J Watkins S VanLandingham M AIDS and older persons: an international perspective. J Acquir Immune Defic Syndr. 2003;33 Suppl 2:S153–65. 10.1097/00126334-200306012-00012 12853864

[13] Moore AR Henry D Experiences of older informal caregivers to people with HIV/AIDS in Lome, Togo. Ageing Int. 2005;30:147–66. 10.1007/s12126-005-1009-8

[14] Knodel J Zimmer Z Kim KS Puch S The effect on elderly parents in Cambodia of losing an adult child to AIDS. Popul Dev Rev. 2007;33:479–500. 10.1111/j.1728-4457.2007.00181.x

[15] Kautz T Bendavid E Bhattacharya J Miller G AIDS and declining support for dependent elderly people in Africa: retrospective analysis using demographic and health surveys. BMJ. 2010;340:c2841. 10.1136/bmj.c2841 20554660 PMC2886852

[16] Gregson S Nyamukapa C Lopman B Mushati P Garnett GP Chandiwana SK Critique of early models of the demographic impact of HIV/AIDS in sub-Saharan Africa based on contemporary empirical data from Zimbabwe. Proc Natl Acad Sci U S A. 2007;104:14586–91. 10.1073/pnas.0611540104 17761795 PMC1961581

[17] Ssengonzi R The impact of HIV/AIDS on the living arrangements and well-being of elderly caregivers in rural Uganda. AIDS Care. 2009;21:309–14. 10.1080/09540120802183461 18780191

[18] Zimmer Z Household composition among elders in sub-Saharan Africa in the context of HIV/AIDS. J Marriage Fam. 2009;71:1086–99. 10.1111/j.1741-3737.2009.00654.x

[19] Kimuna SR Makiwane M Older people as resources in South Africa: Mpumalanga households. J Aging Soc Policy. 2007;19:97–114. 10.1300/J031v19n01_06 17347119

[20] Schatz EJ “Taking care of my own blood”: older women’s relationships to their households in rural South Africa. Scand J Public Health Suppl. 2007;69:147–54. 10.1080/14034950701355676 17676516 PMC2830102

[21] Mugisha J Scholten F Owilla S Naidoo N Seeley J Chatterji S Caregiving responsibilities and burden among older people by HIV status and other determinants in Uganda. AIDS Care. 2013;25:1341–8. 10.1080/09540121.2013.765936 23394785

[22] Kasedde S Doyle AM Seeley JA Ross DA They are not always a burden: older people and child fostering in Uganda during the HIV epidemic. Soc Sci Med. 2014;113:161–8. 10.1016/j.socscimed.2014.05.002 24880658 PMC4065328

[23] Knodel J VanLandingham M Return migration in the context of parental assistance in the AIDS epidemic: the Thai experience. Soc Sci Med. 2003;57:327–42. 10.1016/S0277-9536(02)00361-1 12765712

[24] Angotti N Mojola SA Schatz E Williams JR Gómez-Olivé FX ‘Taking care’ in the age of AIDS: older rural South Africans’ strategies for surviving the HIV epidemic. Cult Health Sex. 2018;20:262–75. 10.1080/13691058.2017.1340670 28741983 PMC5985658

[25] Schatz E Seeley J Gender, ageing and carework in East and Southern Africa: A review. Glob Public Health. 2015;10:1185–200. 10.1080/17441692.2015.1035664 25947225 PMC4888771

[26] UNAIDS. AIDSinfo: global data on HIV epidemiology and response. 2022. Available: https://aidsinfo.unaids.org. Accessed: 1 March 2024.

[27] Bor J Herbst AJ Newell ML Bärnighausen T Increases in adult life expectancy in rural South Africa: valuing the scale-up of HIV treatment. Science. 2013;339:961–5. 10.1126/science.1230413 23430655 PMC3860268

[28] Payne CF Kohler HP The population-level impact of public-sector antiretroviral therapy rollout on adult mortality in rural Malawi. Demogr Res. 2017;36:1081–108. 10.4054/DemRes.2017.36.37 29780281 PMC5959277

[29] Sabin CA Do people with HIV infection have a normal life expectancy in the era of combination antiretroviral therapy? BMC Med. 2013;11:251. 10.1186/1741-7015-11-251 24283830 PMC4220799

[30] Burger C Burger R van Doorslaer E The health impact of free access to antiretroviral therapy in South Africa. Soc Sci Med. 2022;299:114832. 10.1016/j.socscimed.2022.114832 35290814

[31] Schröder H Yapa HM Gómez-Olivé FX Thirumurthy H Seeley J Bärnighausen T Intergenerational spillover effects of antiretroviral therapy in sub-Saharan Africa: a scoping review and future directions for research. BMJ Glob Health. 2023;8:e011079. 10.1136/bmjgh-2022-011079 37068847 PMC10111905

[32] Knodel J The changing impact of the AIDS epidemic on older-age parents in the era of ART: evidence from Thailand. J Cross Cult Gerontol. 2012;27:1–15. 10.1007/s10823-011-9159-5 22205427 PMC3296905

[33] Agincourt - MRC/Wits Rural Public Health and Health Transitions Research Unit 2023. Available: https://www.agincourt.co.za. Accessed: 1 March 2024.

[34] Kahn K Collinson MA Gómez-Olivé FX Mokoena O Twine R Mee P Profile: Agincourt Health and Socio-demographic Surveillance System. Int J Epidemiol. 2012;41:988–1001. 10.1093/ije/dys115 22933647 PMC3429877

[35] Gómez-Olivé FX Montana L Wagner RG Kabudula CW Rohr JK Kahn K Cohort Profile: Health and Ageing in Africa: A Longitudinal Study of an INDEPTH Community in South Africa (HAALSI). Int J Epidemiol. 2018;47:689–90j. 10.1093/ije/dyx247 29325152 PMC6005147

[36] Mojola SA Angotti N Schatz E Houle B “A nowadays disease”: HIV/AIDS and social change in a rural South African community. AJS. 2021;127:950–1000. 10.1086/718234 35967824 PMC9365075

[37] South African Government. Old age pension. 2023. Available: https://www.gov.za/services/social-benefits-retirement-and-old-age/old-age-pension. Accessed: 1 March 2024.

[38] Palinkas LA Horwitz SM Green CA Wisdom JP Duan N Hoagwood K Purposeful Sampling for Qualitative Data Collection and Analysis in Mixed Method Implementation Research. Adm Policy Ment Health. 2015;42:533–44. 10.1007/s10488-013-0528-y 24193818 PMC4012002

[39] Morse JM Determining Sample Size. Qual Health Res. 2000;10:3–5. 10.1177/104973200129118183

[40] McMahon SA Winch PJ Systematic debriefing after qualitative encounters: an essential analysis step in applied qualitative research. BMJ Glob Health. 2018;3:e000837. 10.1136/bmjgh-2018-000837 30233833 PMC6135453

[41] Harris PA Taylor R Thielke R Payne J Gonzalez N Conde JG Research electronic data capture (REDCap)—A metadata-driven methodology and workflow process for providing translational research informatics support. J Biomed Inform. 2009;42:377–81. 10.1016/j.jbi.2008.08.010 18929686 PMC2700030

[42] Green J, Thorogood N. Qualitative Methods for Health Research. Los Angeles, London, New Delhi, Singapore, Whashington DC, Melbourne: SAGE; 2018.

[43] Auerbach CF, Silverstein LB. Qualitative data: an introduction to coding and analysis. New York and London: New York University Press; 2003.

[44] Braun V Clarke V Using thematic analysis in psychology. Qual Res Psychol. 2006;3:77–101. 10.1191/1478088706qp063oa

[45] Kaler A Alibhai A Kipp W Rubaale T Konde-Lule J “Living by the hoe” in the age of treatment: perceptions of household well-being after antiretroviral treatment among family members of persons with AIDS. AIDS Care. 2010;22:509–19. 10.1080/09540120903220287 20162471

[46] Kakinami L de Bruyn G Pronyk P Mohapi L Tshabangu N Moshabela M The impact of highly active antiretroviral therapy on activities of daily living in HIV-infected adults in South Africa. AIDS Behav. 2011;15:823–31. 10.1007/s10461-010-9776-y 20703794

[47] d’Adda G Goldstein M Zivin JG Nangami M Thirumurthy H ARV Treatment and Time Allocation to Household Tasks: Evidence from Kenya. Afr Dev Rev. 2009;21:180–208. 10.1111/j.1467-8268.2009.00207.x 22199461 PMC3244725

[48] Anema A Au-Yeung CG Joffres M Kaida A Vasarhelyi K Kanters S Estimating the impact of expanded access to antiretroviral therapy on maternal, paternal and double orphans in sub-Saharan Africa, 2009-2020. AIDS Res Ther. 2011;8:13. 10.1186/1742-6405-8-13 21385370 PMC3063201

[49] Moore AR Older poor parents who lost an adult child to AIDS in Togo, West Africa: a qualitative study. Omega (Westport). 2007–2008;56:289–304. 10.2190/OM.56.3.e 18300652

[50] Mfundisi C Chiranjan N Rodrigues C Kirchner L Bock P Myer L Availability of antiretroviral therapy is associated with increased uptake of HIV testing services. South African medical journal. 2005;95:483–5. 16156445

[51] Wagner G Ryan G Huynh A Kityo C Mugyenyi P A qualitative exploration of the impact of HIV and ART on social disruption and household continuity in Uganda. Afr J AIDS Res. 2011;10:37–42. 10.2989/16085906.2011.575546 25859618

[52] Siu GE Wight D Seeley J ‘Dented’ and ‘resuscitated’ masculinities: the impact of HIV diagnosis and/or enrolment on antiretroviral treatment on masculine identities in rural eastern Uganda. SAHARA J. 2014;11:211–21. 10.1080/17290376.2014.986516 25444303 PMC4272191

[53] Mojola SA Angotti N Denardo D Schatz E Xavier Gómez Olivé F The end of AIDS? HIV and the new landscape of illness in rural South Africa. Glob Public Health. 2022;17:13–25. 10.1080/17441692.2020.1851743 33290168 PMC8184878

[54] Onoya D Mokhele I Sineke T Mngoma B Moolla A Vujovic M Health provider perspectives on the implementation of the same-day-ART initiation policy in the Gauteng province of South Africa. Health Res Policy Syst. 2021;19:2. 10.1186/s12961-020-00673-y 33407574 PMC7789550

[55] Joint United Nations Programme on HIV/AIDS (UNAIDS). In Danger: UNAIDS Global AIDS Update 2022. 2022. Available: https://www.unaids.org/en/resources/documents/2022/in-danger-global-aids-update. Accessed: 1 March 2024.

[56] African Union. HelpAge International Africa Regional Development Centre. AU policy framework and plan of action on ageing. 2002. Available: https://www.un.org/esa/socdev/ageing/documents/implementation/AUFrameworkBook.pdf Accessed: 1 March 2024.

[57] Bergen N Labonté R “Everything Is Perfect, and We Have No Problems”: Detecting and Limiting Social Desirability Bias in Qualitative Research. Qual Health Res. 2020;30:783–92. 10.1177/1049732319889354 31830860

[58] Global Burden of Disease Health Financing Collaborator Network Spending on health and HIV/AIDS: domestic health spending and development assistance in 188 countries, 1995-2015. Lancet. 2018;391:1799–829. 10.1016/S0140-6736(18)30698-6 29678342 PMC5946845

