# Supplementary material for: “I am adhering to HIV treatment so that I can live to support her”: A qualitative study of upward intergenerational support in South Africa
Source: J Glob Health. 2024 May 10;14:04083. doi: 10.7189/jogh.14.04083 (PMC11082624; doi:10.7189/jogh.14.04083)
Supplement: Online Supplementary Document [file jogh-14-04083-s001.pdf]

Online Supplementary Document

Text S1. Additional context on Older Person's Grant in South Africa 2

Text S2. Types of upward spillover effects from ART to older adults 3

Figure S1. Map of South Africa with study site 12

Figure S2. MRC/Wits Agincourt Unit Health and Demographic Surveillance System study area 13

Table S1. Selected HIV-related characteristics of study participants 14

File S1. Interview guide young or middle-aged respondent (English version) 15

File S2. Interview guide older respondent (English version) 23

File S3. Participant sheet: timeline of support young or middle-aged respondent (English) 30

File S4. Participant sheet: timeline of support older respondent (English) 32

References for Online Supplementary Document 34

Text S1. Additional context on Older Person’s Grant in South Africa

In South Africa, all individuals who are 60 years or older are eligible for the Older Person’s Grant (also called old-age pension grant), which is broadly defined as up to 2,100 South African Rand (about 100 EUR) per month for adults aged 60 or older [1]. The South African Social Security Agency (SASSA) awards Older Person’s Grants based on a means test. Applicants who are single, for example, may not have total assets valued at more than about 1,400,000 South African rand. In our study, 15 older adults (88%) reported receiving an Older Person’s Grant, suggesting that the vast majority of older adults in our sample passed the means test imposed by SASSA (shown in **Table 1** in main text).

Older people who receive Older Person’s Grants often experience a reversal of roles by replacing young or middle-aged family members as the main provider for the family [2-5]. Nevertheless, Older Persons Grants are still relatively small, particularly if several family members depend on the Older Persons Grants and may therefore need to be supplemented by other financial sources such as personal savings or the savings of other family members. Older Person’s Grants may also be more difficult to collect in person in rural areas, such as the Agincourt HDSS area, which are further from the nearest SASSA, and because they require several documents which may be more difficult to provide for individuals from underprivileged settings (such as proof of residence, bank statements, and employment records) [3].

Text S2. Types of upward spillover effects from ART to older adults

1. *Caregiving*

Respondents described how young or middle-aged adults on ART assisted their older family members. Younger adults provided direct care as well as emotional support. They regularly checked on the well-being of older adults, reminded them to take treatment (such as for type 2 diabetes and hypertension in old age), and assisted with treatment collection or accompanied them to clinic appointments. Other types of support included, for example, doing household chores (e.g., cooking, cleaning, washing), fetching water, collecting firewood, and performing other physical work on the property (e.g., fencing, farming, construction work).

> *P: "I go to clean for him [uncle], I cook for him, wash for him and make sure that he has his treatment. I check dates for him to go to the clinic for medication. I sometimes go collect for him the treatment and if they also want him, I go with him. I also collect the food parcels for him. I make sure that where he lives, is a place which is always clean and if he is sick, I make sure I take him to the clinic."*
> *I: "Who else is supporting him?"*
> *P: "It is only me." Female middle-aged respondent (48y) with uncle (81y)*

The extent to which middle-aged respondents on ART provided support to their older family members varied depending on factors such as the health status of older adults, financial resources, living arrangements, and availability of other support networks. Whereas some older respondents were still in good health themselves, other older respondents relied completely on the support of middle-aged family members. For the latter, good health conditions under ART allowed middle-aged adults, for example, to take in their older parents to live with them. An 84-year-old woman who was blind had moved in the house of her adult daughter on ART to be taken care of.

> *P: "I am living with my mother who was living with my brother and his wife, I took her in because she stays alone when they are all at work." Female middle-aged respondent (57y) with mother (84y)*

Good health conditions under ART allowed middle-aged respondents to generally take better care of their older family members in contrast to the time of limited health due to HIV. Some middle-aged respondents directly attributed their ability to provide old-age support to their consistent uptake of ART.

> *I: "How does ART influence your ability to support your mother?"*
> *P: "In a good way, I am able to look after my mother. I do walk on my own to her house and check-up on her and assist her with house chores. I do go to the bushes to fetch firewood for her." Male middle-aged adult (50y) with mother (85y)*

In the long-run, ART was seen as a 'life insurance' for middle-aged adults to be able to provide care for their older family members.

> *P: "My [daughter] adheres to her treatment, so that she can live long to take care of me till I pass on." Female older respondent (85y) with daughter (47y)*

From the perspective of the older generation, ART initiation and gradual recovery among middle-aged adults also reduced the need for older adults to provide direct care to their adult children during HIV-related sickness.

> *P: "By that time when my daughter was sick, I was the one who was supporting her. I was still living in my house at that time, but I had to come here [to my daughter's place] to take care of her, to do everything for her. Now that she has recovered, I am getting all the support from her." Female older respondent (85y) with daughter (47y)*

In addition to that, respondents, particularly older populations, described how ART also indirectly supported older adults by reducing additional HIV-related caregiving burdens or responsibilities to look after orphaned grandchildren.

> *P: "ART is making things easy for me because [name of daughter] will live for her children and I won't have to worry about raising her children." Female older respondent (66y) with daughter (37y)*

Besides the positive upward spillover effects of ART for caregiving from middle-aged adults to older family members, ART also represented a considerable caregiving effort for the older generation. Both middle-aged and older respondents talked about the vital role of older adults in the ART initiation process of their children living with HIV. Older respondents encouraged middle-aged adults to test for HIV, and to start and adhere to treatment. These perceptions indicate possible upward spillover effect of ART (i.e., ART means for older respondents having to encourage and remind younger or middle-aged adults to adhere to treatment with potential additional effort that comes along with ART among middle-aged family members).

> *P: "When [name of son] was diagnosed [with HIV], I was the one who took him to the doctor and even went inside the consultation room with him because I knew that if I don't go in with him, he won't tell me the truth about his illness when he gets out of the consultation room. After the doctor had diagnosed him, I took him to [name of clinic] to be initiated on ART and told the nurses that I will be the one who is going to collect his treatment on his behalf." Female older respondent (60y) with son (40y)*

One older respondent took her sick son back to live with her to be able to support him during the initiation process of ART.

> *P: "[…] his [son's] sickness then got worse that's when I advised him to go to [name of clinic] for ART, after ART collection that particular day he came back to live with me." Female older respondent (77y) with son (50y)*

Older respondents also supported middle-aged family members in the direct uptake of ART, in collecting ART from the clinic or accompanying middle-aged family members to the clinic.

> *P: "I am the one who is collecting treatment for him [son] because he is always at Johannesburg and he does not come home regularly, so when he gets a chance, he comes home to take his treatment and go back to Johannesburg." Female older respondent (60y) with son (40y)*

Despite the described ART-related caregiving effort for older adults, supporting their middle-aged family member's treatment was never described as an additional "burden" among older respondents and hence not a detrimental upward spillover effect.

For middle-aged adults who had started ART early enough to never experience severe HIV-related symptoms, older relatives reported few ART-related changes in their responsibility to provide care to the younger generations, suggesting that the additional burden imposed by caregiving tasks related to ART was perceived to be relatively small.

> *P: "Nothing has been affected by [name of son] HIV treatment. He is still doing the house chores very well. He buys groceries when he has money [...]." Female older respondent (77y) with son (50y)*

Respondents reported an increase of appetite among middle-aged adults after ART initiation.

> *P: "HIV treatment makes them [daughter and granddaughter] to have appetite and eat." Male older respondent (66y) with granddaughter (18y)*

They explained that ART should not be taken "on an empty stomach" and treatment would require a healthy diet. Regaining one's appetite was also appreciated among older adults as it was seen as a sign of recovery of their middle-aged family members. Yet, it involved older adults who needed to support their middle-aged family member's treatment by preparing meals, at least at the onset of ART initiation, and by buying nutritious food to facilitate the uptake of ART (activities which would not be needed in the absence of ART).

> *P: "I provide him [son] with food so that he can be able to take his treatment on a full stomach." Female older respondent (75y) with son (42y)*

Additional supportive quotes:

> *P: "My daughter [niece] is the one who is helping me. She is the one who sees that I am well clothed and fed. She collects my HIV treatment for me and does all things for me." (Male older respondent, 81 years old)*

> *I: "Did your HIV infection affect the support you were giving to your mother?"*
> *P: "What I have realized about this HIV infection is that when you adhere to HIV treatment you will be able to do everything you want to do and if you don't adhere to it, you won't make it to life." (Male middle-aged respondent, 50 years old)*

> *P: "By that time when she [daughter] was sick, I was the one who was supporting her. I was still living in my house that time, but I had to come here [to daughter's place] to take care of her, to do everything for her. Now that she has recovered, I am getting all the support from her." (Female older respondent, 85 years old)*

2. Financial support

Middle-aged respondents on ART assisted their older relatives with money or indirectly by buying items such as groceries or clothes, if financial recourses permitted them to do it. Others helped older adults by doing the traveling and queueing involved with the collection of the Older Person's Grant from the SASSA pay-points.

> *P: "My daughter is the one who is collecting my old age grant on my behalf then she buys groceries for us." Male older respondent (81y) with niece (48y)*

Some middle-aged and older respondents mentioned several changes in financial support to older adults which they attributed to consistent ART use among middle-aged adults. Middle-aged respondents who had suffered severe HIV-related symptoms, were described as being unable to financially support their older family members during sickness.

> *I: "Has your HIV diagnosis affected the support you were giving to your parents?"*
> *P: "It affected them a lot because I have not been working for some time now and most of the things in the house, I was the one who was providing, so since I am not working, I am unable to support them. [...] I was supporting them by giving them money to buy groceries and other things that are not in the house." Male middle-aged respondent, (40y) with father (68y)*

Although recovered (or maintaining good health) middle-aged adults on ART could support their older household and family members by sending money or by purchasing household items, older adults normally were reported to live from their old age pension money and middle-aged adults were commonly unemployed. Thus, healthy unemployed middle-aged respondents, despite being on ART, still had limited financial resources and only few were able to financially support their older family members.

> *P: "I have vowed to support my parents in any way I can afford, so the [HIV] treatment is giving me a chance to live, so that I can support them. [...] if I have that small amount with me, I do give them to buy themselves a soft drink. They are receiving their old age grant with which they are supporting themselves with but when I visit, I give them something from my pocket." Male middle-aged respondent (53y) with father (81y)*

As a result, many families and households depended on so called "pooling" grants, commonly shared money received from governmental grants such as the Child Support Grant and Older Person's Grant.

> *P: "We are poor. My children do not work, we survive with my old age grant. If they had jobs maybe they would have improved the house, we are living in, but poverty is following us. I am the breadwinner of the family and things are being taken care of by me." Female older respondent (68y) with son (37y)*

From the perspective of the older generation, the older respondents in our sample also noted changes in expenditures resulting from the availability of ART among middle-aged adults. For example, older respondents experienced a reduction in HIV-related expenditures such as on visits to clinics or traditional healers.

> *P: "She [mother] was supporting me at the time I was very sick; she would give me money to go the clinic when I had no money from her old age grant." Male middle-aged respondent (50y) with mother (85y)*

Moreover, the consumption of traditional medicines to address HIV-related symptoms among middle-aged adults was reduced once these middle-aged adults recovered after the initiation of ART.

> *P: "[...] I took her [daughter] to different traditional healers, but it did not help, and I was losing a lot of money. I then took her to the clinic where she was diagnosed with HIV. Then the nurses told us to stop feeding her with traditional medicines and to give her ART. That is when she started taking ART, till to date." Female older respondent (66y) with daughter (37y)*

Despite broadly beneficial changes in financial support for older adults, initiating and maintaining ART among middle-aged adults also entailed financial costs. With relatively limited financial recourses, which is the case for most households located in the HDSS area, raising funds to pay for transport for treatment collection or attending clinic appointments was an important concern across generations. Following ART initiation, several middle-aged respondents on ART had to travel "far" to the next clinic to collect their treatment. Middle-aged respondents therefore often relied on older family members to provide the financial means for their transport to the clinic to pick up ART.

> *P: "She [mother] sometimes gives me money for transport to the clinic for collection or ART." Male middle-aged adult (42y) with mother (75y)*

One older respondent talked about the difficulties associated with providing money for her grandson.

> *P: "There were times, I did not have money to take him [grandson] to the clinic for his HIV treatment collection then I had to borrow and pay when I received my old-age grant." Female older respondent (83y) with grandson (19y)*

Additional supportive quotes:

> *I: "Does she [daughter-in-law] support you financially?"*
> *P: "Yes, she sometimes gives me money when I want to go to the clinic."*
> *I: "Does she provide you with house assets or groceries?"*
> *P: "Yes, as I have said that when she receives her child support grants, she does buy some groceries for us to eat, to say, she is the one who is supporting me more than my children." (Female older respondent, 60 years old)*

> *P: "When I tested positive, I was unemployed, and they [older family members] supported me financially when I needed to collect my medication and I was collecting them far." (Female middle-aged respondent, 48 years old)*

> *I: "So is the grant enough to sustain you?"*
> *P: "Not at all. Because I have debts. [...] from people. Because I would borrow money, at the time these children were sick [due to HIV] for transportation to the hospital." (Female older respondent, 85 years old)*

3. Health outcomes

ART among middle-aged adults was reported to have positive upward spillover effects on the health of older adults, including for their physical and mental health. The initiation of and adherence to ART among middle-aged adults improved the subjective well-being of older adults who were worried about the health of their middle-aged adults diagnosed with HIV.

> *I: "Did your HIV treatment have positive or negative consequences on your mother?"*
> *P: "I can say that they are positive because when she sees me in this good condition now, she is happy and stress-free. So, if I was not adhering, I would have died or I would still be sick even now and that would cause illness to her too." Male middle-aged respondent (50y) with mother (85y)*

Consistently, one of the most common topics addressed when older respondents were asked how they were affected by the uptake of ART among their middle-aged family members was “to worry less about their adult children’s health”.

> *P: “I was happy that he [son] was finally diagnosed [with HIV] and he is taking treatment, then he will live long.” Female older respondent (75y) with son (42y)*

The large impact of ART on the subjective well-being of older adults was evident in those cases in which HIV-related sickness among family members had caused severe suffering. Older respondents described the fear of losing a child to AIDS and the great relief upon seeing their sick child initiate ART and recover their health.

> *P: “It [son’s HIV diagnosis] has affected me a lot, it has hurt me a lot but its better now because [name of son] has a treatment for this virus if not I would have lost him along with his first wife. Female older respondent (68y) with son (37y)*

Nevertheless, while older adults worried less about their middle-aged relative’s HIV diagnosis, by observing the beneficial health effects of ART (effectively treating HIV and preventing AIDS), they did worry about their adherence to treatment.

> *I: “How has ART affected your older family members especially you mother? And did it affect her in a positive and negative way?”*
> *P: “It has affected her in a negative way, because she worries a lot when she sees me losing weight and she would enquire if I am still taking treatment, I always tell her a person can lose weight by also overthinking things. It affects her a lot since she worries a lot.” Male middle-aged adult (51y) with mother (75y)*

Moreover, seeing their adult children go out drinking alcohol and return late at night led older adults to worry and advise their children to take their medication with them in their pockets when going out at night.

> *P: “If you stop [taking ART], they say you will meet with Jesus and since he [son] drinks alcohol very much, we told him to put the treatment in his pocket. He will take it wherever he drinks.” Female older respondent (71y) with son (31y)*

Few middle-aged adults decided against disclosing their HIV diagnosis to their older family members to avoid causing trouble and potentially negatively impacting their health. Staying healthy thanks to ART enabled middle-aged adults to keep their HIV diagnosis a “secret”.

> *P: “I was told not to disclose my HIV status to my mother because she has high blood pressure, and she might get worried and die if she can know of my HIV status.” Female middle-aged respondent (57y) with mother (84y)*

Based on a general perceived risk in the community that HIV can be transmitted during activities of direct caregiving such as bathing, assisting with eating, or clothing, the following female middle-aged respondent pointed out to a perceived benefit of ART for older adult’s health by reducing the risk of HIV transmission during such caregiving activities.

> *P: “There was a time my mother was sick I used plastic hand gloves bath her, feed her to protect her from being infected. We also took my mother HIV tests because I am the one who is doing most of her things. There was my cousin who was close to my mother the time my mother still had her site. She was HIV positive, and she stopped taking ART then she became very sick, and my mother was taking care of her, she died of AIDS there. We took my mother for HIV testing just in case she*

got infected while I was taking care of her." Female middle-aged respondent (57y) with mother (84y)

Furthermore, one middle-aged respondent mentioned that the regular ART uptake among middle-aged adults could motivate older family members to collect their own treatment (not necessarily HIV medication) at the clinic.

P: "When we started drinking [HIV medication] even them [older family members] when they started feeling pains, they continued going to hospital to collect the treatment, they were being motivated by seeing us taking ART, [...]." Female middle-aged respondent (43y)

Similarly, a male older respondent who himself was on ART saw a benefit in living together with a wife who is taking the same treatment since they could support each other.

P: "A widower should get married to a widow, so much with a HIV person should get married to another HIV person so that you can both remind each other when it's time to take ART [...]." Male older respondent (60y) with wife (43y)

Additional supportive quotes:

P: "What has made things not to change is because I did not stress about him [son] being HIV positive. Instead, I was more interested on the part that he is taking the treatment or not. I have seen many people who are HIV positive and who are adhering to treatment living long without any complications. So, I took it to heart that my son will live too if he adheres to treatment. I was not affected in any way." (Female older respondent (75 years old)

I: "Has ART to your daughter affected you in any way?"
P: "No, I am seeing positive results because she is adhering to treatment and that makes me happy. My daughter is a fighter and I believe she is still here because she knows that if she would die, I will suffer. She is my strength." (Female older respondent, 85 years old)

P: "If he [son] doesn't stop taking his treatment, he would live. But because he also enjoys drinking alcohol, he will stop the treatment."
I: "So he often drinks?"
P: "Yes, he does. I even ask his wife whether he takes his treatment accordingly. And my wish is only if he can just stop drinking." (Female older respondent, 85 years old)

4. *Living arrangements and relationships*

Consistent uptake of ART among middle-aged adults had the potential to restore and prevent changes to the living arrangements and household relationships of older adults. For instance, an older respondent who had fought for the life of her son suffering from severe HIV-related sickness prior to his initiation on ART, reported ART to be the premise for harmonious family relationships.

P: "Our relationship stays good as long as those who must take [HIV] treatment take treatment." Female older respondent (75y) with son (51y)

Respondents described discussing HIV and AIDS with care among family members, and rarely undertaking discussions beyond the technical details of treatment adherence (e.g., reminding a

family member to take treatment or to collect ART at a health facility). Several older respondents had experienced or feared disrupted family relationships because of HIV.

I: "Has HIV treatment been a topic openly discussed in your family?"
P: "I do talk about it with [name of son 1] but not with [name of son 2] and my daughter in-law because I think it might cause havoc in the family." Female older respondent (60y)

ART allowed middle-aged adults to stay healthy and thus to keep their HIV diagnosis and treatment a secret from family members intending to prevent conflicts within families.

P: "We [family] suggested that [name of son 1] should not know about [name of son 2] HIV status because when he is drunk, he will disrespect [name of son 2]. [Name of son 1] does know about [name of son 2] HIV status we are the ones that go and collect son's treatment, by keeping this a secret I want my family to be in peace." Female older respondent (60y) with son 1 (42y) and son 2 (40y)

In addition, the topic of HIV and ART may have the potential to strengthen intergenerational family ties since many respondents described the importance of discussing HIV and ART within the family as well as comforting each other.

P: "[…] my son couldn't disclose his [HIV] status. After we saw that he was sick we grouped together with his sisters and asked him to take [HIV] treatment until he agreed, and we were happy when he started the treatment." Female older respondent (75y) with son (51y)

Reports from the small sample of three older respondents who were treated with ART themselves indicate that these older adults were more understanding when it came to the topic of middle-aged family members or spouse diagnosed with HIV and on ART. Their relationships were not disrupted by HIV but on the contrary family members and partners strongly supported each other regarding ART initiation and adherence. For example, an older respondent disclosed her status to her children to facilitate their coping process with the HIV diagnosis.

P: "I disclosed my HIV status to her [daughter] to make it easy for to accept the situation." Female older respondent (65y) with five children on ART

Additional supportive quotes:

I: "What did the HIV treatment change in your family?"
P: "It did not change anything; everything is still the same." (Female older respondent, 77 years old)

P: "[…] when we stay with them [older family members] we communicate about it [HIV and ART], when someone is sick do not show each other with fingers. So, others die because of leaving the treatment, even the child of my sister-in-law died because of stopping the treatment, they said he was bewitched. We talk about it because of it. When you stop [taking ART] you are going to die." (Female middle-aged respondent, 43 years old)

P: "His [son's] HIV infection had not separated my family members; we still treat each other with respect to maintain each other's dignity. Both my sons do adhere to HIV treatment, if I can show you [name of son 1] you can also see that he is adhering to HIV treatment because he is fatty and healthy, he went to collect his HIV treatment a day before yesterday and [name of son 2] went today and he is adhering to HIV treatment." (Female older respondent, 77 years old)

5. *Stigma and reputation*

Respondents explained that the key to avoiding adverse community reactions was to stay healthy since sickness would lead to others speculating about ones HIV-status. As a result, controlling HIV with the help of ART enabled the possibility to prevent stigmatization by community members.

> *P: "[...] I did not want to get very sick before taking ART as I was avoiding being stigmatized by community members because they will see me at a very bad condition. [...] Community members will not know your HIV status when you start ART while you are in a good condition but when you wait till you get very sick people will know your HIV status." Male older respondent (60y)*

Thus, discussions surrounding HIV and AIDS as well as ART among middle-aged adults primarily occurred within the confines of the family, rarely extending to the broader community or workplace colleagues. Almost all young or middle-aged respondents (97%) disclosed their HIV-status to at least one family member.

Since the middle-aged adult's HIV and treatment status was usually kept as a family's secret, the reputation of the family including older adults was not affected.

> *P: "Most people are taking HIV treatment and I don't go around talking about my son's HIV status. I had taken it like any other disease and my reputation in the community has not changed a bit." Female older respondent (77y) with son (48y)*

Additional supportive quotes:

> *I: "Do you think ART improves the reputation of you and your wife in this community?"*
> *P: "Your reputation in the community changes when people know your HIV status and they start to talk about you when they are out there drinking alcohol, so to us that the community does not know our HIV status [thanks to ART], our reputation is still the same." (Male older respondent, 60 years old)*

> *I: "Do you think ART has improved the reputation of your daughter in-law?"*
> *P: "Yes, because if she was not taking ART, she would have gotten sick and as you know people tend to detect people's HIV status, the whole villagers would be talking about her. Now I am worried because one of the villagers is the one who gave me [name of son] ART, I am worried she will tell the villagers about my son's HIV status." (Female older respondent, 60 years old)*

> *I: "Does the HIV diagnosis among your children changed how the community sees them?"*
> *P: Nothing changed, even to me as their mother nothing changed, we are still the same as they know us because they don't know that we are HIV positive and on treatment. There are no changes on how they see you if they don't know, it only changes when community members know. If you can tell someone about your family issues, they will go out there to the community to tell them and protect their own issues to be on public." (Female older respondent, 65 years old)*

Figure S1. Map of South Africa with study site

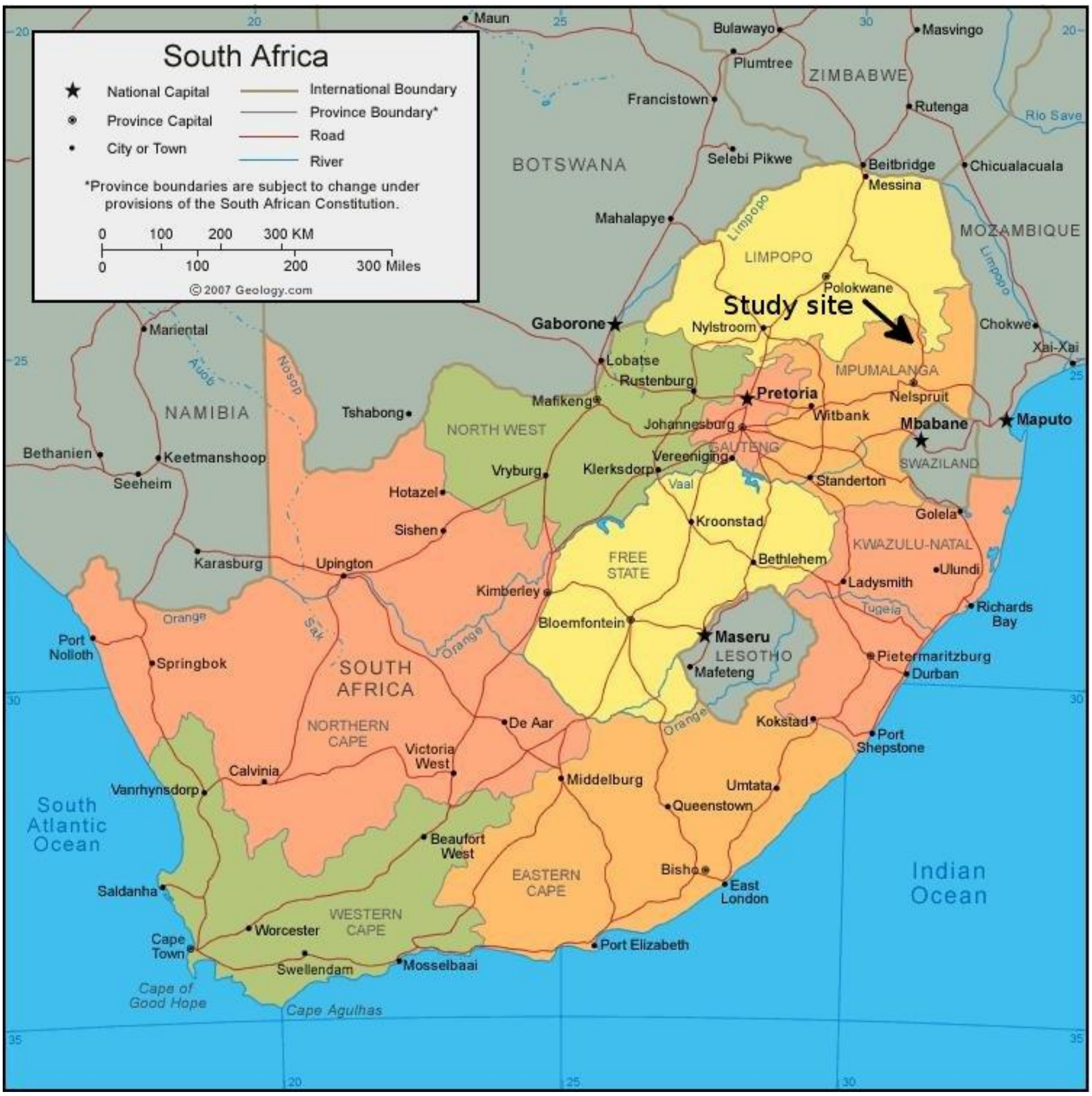

Notes: Figure shows a map with the location of the study site (as indicated by the black arrow in the figure) in the north of Mpumalanga Province in South Africa. Source: www.agincourt.co.za.

Figure S2. MRC/Wits Agincourt Unit Health and Demographic Surveillance System study area

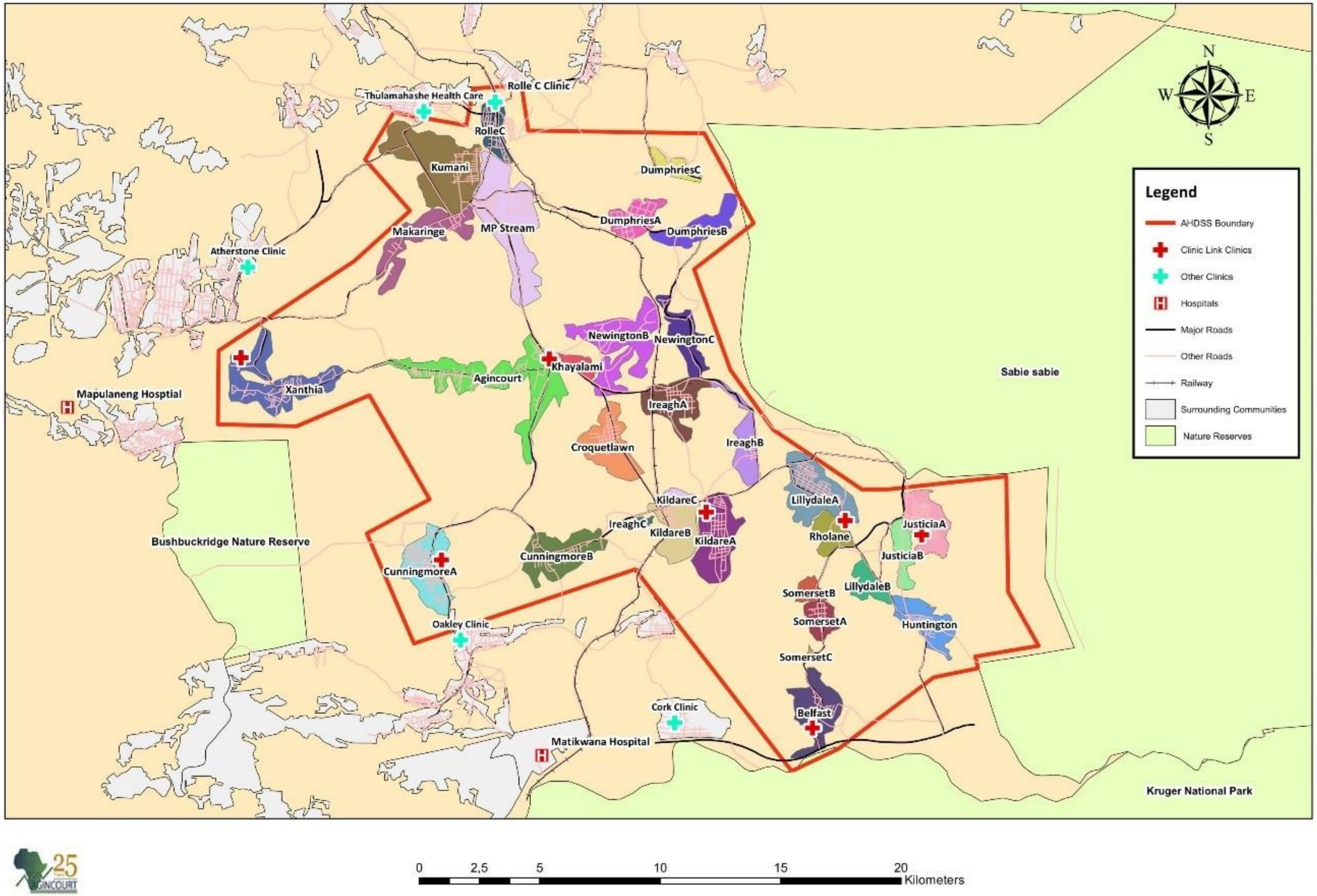

Notes: Figure shows a map with the Agincourt Health and Demographic Surveillance System (HDSS) study area as of 2020. Source: www.agincourt.co.za

Table S1. Selected HIV-related characteristics of study participants

Subsample	**Young or middle-aged respondents (n=29)**	**Old-age respondents (n=17)**	**Pooled sample (n=46)**
Date of HIV diagnosis			
2000-2005	2 (7%)	1 (6%)	3 (7%)
2006-2010	4 (14%)	1 (6%)	5 (11%)
2011-2015	12 (41%)	0 (0%)	12 (26%)
2016 or later	10 (34%)	0 (0%)	10 (22%)
At birth	1 (3%)	0 (0%)	1 (2%)
Unknown	0 (0%)	1 (6%)	1 (2%)
Not applicable (HIV-)	0 (0%)	14 (82%)	14 (30%)
Start of HIV treatment since diagnosis			
Same year	24 (83%)	2 (12%)	26 (57%)
One year after	2 (7%)	1 (6%)	3 (7%)
≥2 years after	3 (10%)	0 (0%)	3 (7%)
Not applicable (HIV-)	0 (0%)	14 (82%)	14 (30%)
HIV-related symptoms			
Never	13 (45%)	1 (6%)	14 (30%)
Mild	9 (31%)	1 (6%)	10 (22%)
Severe	7 (24%)	1 (6%)	8 (17%)
Not applicable (HIV-)	0 (0%)	14 (82%)	14 (30%)
Reason for HIV testing			
Voluntary	4 (14%)	1 (6%)	5 (11%)
Sickness	14 (48%)	2 (12%)	16 (35%)
Clinic visit (family planning, antenatal care)	5 (17%)	0 (0%)	5 (11%)
Testing at work	3 (10%)	0 (0%)	3 (7%)
Other reason	3 (10%)	0 (0%)	3 (7%)
Not applicable (HIV-)	0 (0%)	14 (82%)	14 (30%)
Disclosure of HIV status			
To at least one family member	28 (97%)	3 (18%)	31 (67%)
Did not disclose status	1 (3%)	0 (0%)	1 (2%)
Not applicable (HIV-)	0 (0%)	14 (82%)	14 (30%)

Notes: Table shows selected clinical characteristics of all study participants, separately for young or middle-aged and older adults (*n*=46). Young or middle-aged was defined as ages 18-59 years old and older age was defined as ages 60 years or older. HIV-related symptoms were defined as HIV-related symptoms ever experienced and included slight weakness, dizziness, skin rash, headache, coughing, loss of appetite ("mild") and severe manifestation of symptoms such as weakness, weight loss, diarrhea, and comorbidities such as tuberculosis ("severe"). Data on HIV were extracted from patient records from clinics in the Agincourt HDSS area and complemented by data from the interviews as needed.

File S1. Interview guide young or middle-aged respondent (English version)

Interview Guide

Introduction

1. Good day, I am Mr/Mrs ______________________from the MRC/Wits-Agincourt Research Unit. *(Introduction of all persons present)*
2. Thank you very much for your participation in this study.
3. I am here to learn from **you** because **you** are the **expert on your experiences and views**. I am interested in **everything** you like to share with me.
4. With your help we want to **improve the living circumstances for families** that have been affected by HIV.
5. Just as a reminder our interview will probably last around **45-90 minutes**.
6. As we went over in the consent form, all the information you provide will be kept **confidential**. Everything you say **cannot** be linked to your personal information.
7. We are an independent team of researchers. **Nothing** you say will have an impact on you and your family.
8. I will place this voice recorder in front of us. You do **not** have to worry about this recorder we can just focus on the conversation between the two of us.
9. Do you have any questions before we begin?
10. May I start the recording?

[Start recording]

Interview guide

Good day, thank you for participating today!

For my first questions I would like to ask you about your home and your living circumstances.

**A. Home and living circumstances**
**1. Could you please start telling me about your home?**<br>a. […] please start with what comes first to your mind.<br>b. You can tell me everything that comes to your mind when you think about your home.<br>c. Could you please describe me the home where you live in?
**2. <u>Where</u> do you live?** *(if not already covered in 1)*<br>*Possible answers:*<br>*i. Rural area, urban area, possible other circumstances*<br>*ii. House, apartment, single room, shared living-space, possible other circumstances*<br>*a. Where do you live in the house?*<br>*Possible answers:*<br>*i. Own room, shared room, other possible circumstances*

B. Occupation and living situation
**3. What is your current occupation?**<br>a. How do you spend your day?<br>b. What do you do for living?<br>*Possible answers:*<br>*i. Employed, working, retired, other possible circumstances*
**4. Can you support yourself?**<br>*Possible answers:*<br>*i. Sufficient/insufficient income/recourses*
**5. How do you support yourself?**<br>a. From which resources do you live?<br>b. Are you economically independent, partially independent, or dependent?<br>*Possible answers:*<br>*i. Earnings, savings, assets,*<br>*ii. Subsistence farming*<br>*iii. Social security (e.g. old-age pension)*<br>*iv. Wealth transfers from family or friends (if family, who?)*<br>v. *Other income source*
**6. How would you describe your living situation?**<br>a. Are you happy with your current living situation?<br>b. Is there anything you have to worry about in your daily life?

Thank you for sharing about your current occupation. Now I would like to ask you some questions to the people you live with.

C. Household structure
**7. With whom do you live together?**<br>*a.* With how many people do you share your house/apartment/room?<br>*i.* How many people live in this household?<br>*b. Who is living with you in the same household?*<br>*Possible answers:*<br>i. *Family, friends, others, no household members, other possible circumstances*
**8. How old are the older household members you live with?**
**9. Who is the head of the household?**
**10. Please tell me more about <u>living together</u> with your family/household members.**<br>a. How is your relationship to your family or household members?<br>b. What do you like about living together with your family/household members?<br>c. What do you not like about living together with your family/household members?<br>d. Please tell me about your interaction with your family/household members.<br>e. With whom do you spend most of your time?<br>*f.* Who do you talk to a lot?
**11. How would you describe your role within the household/family?**<br>a. Where do you see yourself in the household/family?<br>b. What do you do in the house?<br>*Possible answers:*<br>*i. Occupation at home: cooking, etc.*<br>ii. *Caregiver and/or care-receiver*<br>iii. *Dependent vs. independent*<br>iv. *Possible other roles*

D. Family structure
**12. Are your parents still living?** *[If no parents and no older household members (≥60 years old), exclusion criteria]*<br>a. Are these your biological parents?<br>b. Are your 'social parents' still alive?<br>*Possible answers:*<br>i. E.g., adoptive parents, stepparents?
**13. How old is/are your parent(s)?** *[Younger than 60 years old, exclusion criteria]*<br>a. Where is/are your parent(s) living?<br>b. Are your parents married?<br>c. What is their educational level?<br>d. How often do you see your parent(s)?
**14. How many children do you have?**<br>a. Please tell me more about your child/children.<br>b. What is the age of each of your child/children?<br>c. How many of your children are living with you in the same house?<br>d. How many of your children are living at another place?
**15. Do you have to care for younger children 0-17 years old in the house?**<br>a. If yes, how? Please describe.
**16. Do you have grandchildren?**<br>a. If yes, how many?<br>b. Are the grandchildren living with you in the same house?

E. Working-age and support
**17. Do you receive support from somebody of your family or household?**<br>a. If yes: from whom?<br>b. If no: why not?
**18. Do you receive support by anybody outside the family or household?**<br>a. If yes: from whom?<br>b. If no: why not?
**19. Are you supporting anybody regularly?**<br>a. Who are you supporting regularly?<br>b. How? Financially, socially, or by other means? Please describe.<br>c. If financially, how much money do you provide e.g., per month?<br>i. What percentage of your monthly earnings do you provide?<br>ii. If socially, do you provide or support e.g., direct care to someone?

Thank you for your answers. I would like to learn more about your parents/your older household members.

F. Old-age and support
**20. How do your parent(s)/older household members do?** *[In case of no parents, focus on older household members (≥60 years old)]*<br>a. How would you describe their well-being?<br>b. How would you describe their living-situation?
**21. How is your relationship to your parent(s)/older household members?**<br>a. How often do you see your parent(s)/older household members?<br>b. How often do you interact with your parent(s)/older household members?<br>c. How much time do you spend with your parent(s)/older household members?<br>d. How do you spend your time with your parent(s)/older household members?<br>*Possible answers:*

i. *Personally, phone-call, other possible circumstances*

22. Is/are your parent(s)/older household members dependent on support?

a. What kind of support do they need?
Possible answers:
i. Health care, financial, social, other possible support?

b. Who is supporting them?

23. Are you supporting your parents(s)/older household members?

a. How do you support them?

24. Are your parent(s) older household members supporting you?

a. What kind of support do you receive?
Possible answers:
i. Health care, financial, social, other possible support?

HIV and HIV treatment have been a topic in your life. I would like to encourage you to tell me as much as you want to about your diagnosis and treatment. **Please keep in mind that anything you say will be kept confidential.**

G. Working-age and HIV

25. Do you remember the time that you were diagnosed with HIV?

a. Please walk me through the process of you getting tested HIV positive.
b. How long is it ago?
c. How was your health status at the time?
d. When did you start with HIV treatment?

26. Did the HIV diagnosis change anything in <u>your</u> life?

a. Did the disease have an effect on your life?
  i. Positive consequences?
  ii. Negative consequences?
b. How did the HIV infection affect your:
  i. Physical well-being?
  ii. Psychological/mental well-being?
c. How does the diagnosis affect your:
  i. Health?
  ii. Work/occupation?
  iii. Economic situation?
  iv. Caring for your parent(s)?
  v. Caring for your child(ren)?
  vi. Reputation?
  vii. Social environment?
  viii. Other effects (please specify)?

27. Which effect does/did your HIV infection have on your family/household?

a. Please walk me through the process of being tested HIV positive and the perceived consequences this had for your family.
b. How did you and your family go through the process of having to deal with HIV?
c. How does the diagnosis affect the relationship within your family?
d. How does the diagnosis change your living together with household members?
e. How did the infection/disease affect household support structures?

28. How does your family deal with your HIV diagnosis?

a. How do you deal with the topic of HIV in your family?
b. Is HIV an openly discussed topic in your family?
c. Does everybody in the family know about your HIV infection?

29. Did <u>your</u> HIV related disease (e.g., AIDS) have an impact on your older parent(s) and/or other older household members?

a. Did HIV infection affect you supporting your parent(s)/older household members?<br>b. How did HIV affect your ability to support your parents?<br>c. Did HIV infection affect the support you receive from your parent(s)/older household members?

Thank you for talking with me about your HIV diagnosis. Led us continue to look at the HIV treatment, the so-called ‘Antiretroviral therapy’ or in short ‘ART’.

**H. Working-age and ART**
**30. Do you remember the first time you heard about Antiretroviral therapy/HIV treatment?**<br>a. When was the first time you heard of ART?<br>b. When did you start with Antiretroviral therapy?<br>c. Could you please explain your treatment status?<br>i. Is your viral load detectable?<br>*ii. Virally suppressed vs. not virally suppressed*<br>d. How often do you take Antiretroviral therapy?<br>e. Would you say you are compliant to HIV treatment?<br>i. Do you take your medication regularly?<br>ii. Do you attend nurses/doctor appointments regularly?<br>f. Has ART been consistently available to you?<br>g. Do you still have HIV-related symptoms?<br>h. Do you feel well informed about Antiretroviral therapy?
**31. What do you think of ART?**<br>a. What are major benefits?<br>b. What are major disadvantages?<br>c. What are your concerns?
**32. What does/did HIV treatment change in <u>your</u> life?**<br>*a. Positive consequences?*<br>*b. Negative consequences?*<br>*c.* How does/did HIV treatment influence:<br>*i. Your physical health?*<br>*ii. Your mental health?*<br>*iii. Your ability to go to work?*<br>*iv. Your economic situation*<br>*v. Your reputation?*<br>*vi. Your social environment?*<br>*vii. Your support structures?*<br>*viii. Your perceptions of "modern medicine" (vis-a-vis traditional healers)?*<br>*ix. Your health-seeking behavior (clinic vs. traditional healer)?*<br>*x. Other effects (please specify)*

**I. Old-age and ART**
**33. Did <u>your</u> HIV treatment have an impact on <u>your older parent(s) and/or older household members</u>?**<br>*a. Positive consequences for older people?*<br>*b. Negative consequences for older people?*<br>c. How does ART influence:<br>*i. Your ability to support your older family/household members?*<br>*ii. Your ability to send/give them money?*<br>*iii. Your ability to provide direct care to them?*<br>*iv. Your ability to support them to access the formal health system?*

i. *E.g., providing transport to the clinic; buying insurance; paying out-of-pocket fees for primary care or clinic costs; other possible circumstances.*<br>v. *Any other effects? Please explain.*
**34. Do you feel that the overall consequences of ART are positive or negative for you?**<br>a. …for your family? If so, why?<br>b. …for your broader community? If so, why?<br>c. …for your status? If so, why?<br>d. …for your social environment? If so, why?
**35. Do you think that further testing for HIV in your community would be good?**
**36. Would further expanding access to HIV treatment in your community have societal benefits or disadvantages?**<br>a. If so, please explain.

I will now give you a piece of paper and a pencil. Please make a mark on the line according to the questions that I will ask you. Please share your thoughts with me while working on the questions.

**J. Timeline of support**
**37. Present**<br>a. How much do you support your older family member(s) today?
**38. Past**<br>a. How much did you support your older family members(s) in the past (before your HIV diagnosis)?
**39. HIV diagnosis**<br>a. How much did you support your older family member(s) at the time of your HIV diagnosis?
**40. HIV treatment**<br>a. How much did you support your older family member(s) at the time you started taking HIV treatment?
**41. Other event**<br>a. Is there an event that changed the way you supported your older family member(s)?<br>b. How much did you support your older family member(s) at the time of this event?

**K. HIV and ART and stigma/disclosure**
**42. Do you think people keep the HIV diagnosis a secret?**<br>If so, why do you think people keep HIV a secret and do not talk about it?
**43. Do you think that HIV treatment can improve the stigma of HIV and AIDS?**
**44. Do you think that men and women do things differently with respect to HIV and treatment?**<br>If so, what do you think they are doing differently?<br>a. Do they differ in:<br>i. HIV-testing?<br>ii. Health seeking behaviour (going to clinic)?<br>iii. Disclosing the status?<br>iv. Talking freely about related topic?<br>v. Adhering to HIV treatment?<br>vi. Talking open about HIV treatment?

**L. Conclusion**

45. Is there a question that you think of important and which I have not asked during this conversation?
   a. Is there anything else you would like to share to the topic that hasn't been voiced?

Before we come to an end, I would like to check a list with you to see if I have asked you all important questions. [O*nly complete what has not been covered, otherwise skip section!]*

M. Personal data

46. Respondent's

a. Personal data
   i. Age
   ii. Gender
   iii. Marital status/Partnership
   iv. Schooling level (as either e.g., no formal schooling; some primary; primary completed; some secondary; or secondary schooling or higher)

b. Family
   i. Number of children
   ii. Age of children
   iii. Children's place of residence
   iv. Age of grandchildren
   v. Number of grandchildren
   vi. Biological/social parents alive/dead
   vii. Age of parents

c. Home and living circumstances
   i. Place of residence
   ii. Home (How does respondent live? House, apartment, single room, shared living-space, possible other living circumstances)
   iii. Head of household
   iv. Exact number of household members
      1. Partner
      2. Parents
      3. Children
      4. Grandchildren
      5. Friends
      6. Others *(please specify)*

d. Work life/Retirement status
   i. Current occupation
   ii. Work status/retirement status
   iii. Economic support structures
   iv. Economic situation: Earnings, savings, assets
   v. Social security (e.g. old-age pension)

e. HIV status and HIV treatment
   i. Date of HIV diagnosis
   ii. Date of start of HIV treatment
   iii. HIV treatment status (Not virally suppressed, virally suppressed)
   iv. Date of start with ART
   v. Compliance to ART
      1. Attending appointments regularly?
      2. Taking the HIV treatment regularly?
   vi. Number of appointments at health clinics
   vii. HIV-related symptoms
   viii. ART-related symptoms

Thank you very much for sharing all your answers with me. We are at the end of the interview. I will stop the recording.

[Stop recording]

Do you have questions concerning the interview or the study?

Thank you for your participation!

File S2. Interview guide older respondent (English version)

Interview Guide

Introduction

1. Good day, I am Mr/Mrs ______________________from the MRC/Wits-Agincourt Research Unit. *(Introduction of all persons present)*
2. Thank you very much for your participation in this study.
3. I am here to learn from **you** because **you** are the **expert on your experiences and views**. I am interested in **everything** you like to share with me.
4. With your help we want to **improve the living circumstances for families** that have been affected by HIV.
5. Just as a reminder our interview will probably last around **45-90 minutes**.
6. As we went over in the consent form, all the information you provide will be kept **confidential**. Everything you say **cannot** be linked to your personal information.
7. We are an independent team of researchers. **Nothing** you say will have an impact on you and your family.
8. I will place this voice recorder in front of us. You do **not** have to worry about this recorder we can just focus on the conversation between the two of us.
9. Do you have any questions before we begin?
10. May I start the recording?

[Start recording]

Interview guide

Good day thank you for participating today!

For my first questions I would like to ask you about your home and your living circumstances.

**A. Home and living circumstances**
**1. How would you describe your living situation?**<br>a. […] please start with what comes first to your mind.<br>b. You can tell me everything that comes to your mind when you think about your home.<br>c. Could you please describe me the home where you live in?
**2. <u>Where</u> do you live?** *(if not already covered in A1)*<br>*Possible answers:*<br>*i. Rural area, urban area, possible other circumstances*<br>*ii. House, apartment, single room, shared living-space, possible other circumstances*

Thank you for your answers. Let us move on and talk more about your home and family.

**B. Household structure**
**3. With whom do you usually live together?**

c. With how many people do you share your house/apartment/room?<br>i. How many people live in this household?<br>*d.* Who is living with you in the same household?<br>*Possible answers:*<br>i. *Family, friends, others, no household members, other possible circumstances*
**4. Who is the head of the household?**
**5. How would you describe your role within the family/household?**<br>c. Where do you see yourself in the family?<br>d. What do you do in the house?<br>*Possible answers:*<br>*i. Occupation at home: cooking, etc.*<br>ii. *Caregiver and/or care-receiver*<br>iii. *Dependent vs. independent*<br>iv. *Possible other roles*

**C. Family structure**
**6. Is anybody of your parents still living?**<br>a. If yes, how many?<br>b. If yes, how old are they?
**7. Do you have grandchildren?**<br>a. If yes, how many?<br>b. If yes, how old are they<br>c. Are the grandchildren living with you in the same house?
**8. How many children do you have?**<br>a. What is the age of each of your child/children?
**9. Please tell me more about your child/children.**<br>a. With how many of them do you generally co-reside?<br>b. How many of your children are living with you in the same house?<br>c. How many of your children are living at another place?<br>*d.* How many of them migrate for work but return regularly to your household?<br>*e. Possible other circumstances?*

**D. Old age and health**
**10. How old are you?** *[Minor than 60 years old, exclusion for older respondent]*
**11. Are you in good health?**<br>a. If not, please precise…<br>b. What are your major health concerns?<br>c. Do you have chronic diseases?
**12. What is your HIV status?**<br>a. Are you HIV positive?<br>b. If HIV positive, are you on HIV treatment?<br>c. If HIV positive, do you know if your viral load is suppressed?<br>d. If HIV positive, how does the infection influence your well-being?<br>e. If HIV positive and on ART, how does ART influence your well-being?
**13. What is most important for you to remain in good health?**

**E. Occupation and living situation**
**14. What is your current occupation?**<br>a. How do you spend your day?<br>b. What do you do for living?<br>*Possible answers:*

i. *Employed, working, retired, other possible circumstances*

15. Can you support yourself?

Possible answers:

i. *Sufficient/insufficient income/recourses*

16. How do you support yourself?

a. From which resources do you live?
b. Are you economically independent, partially independent, or dependent?

Possible answers:

i. *Earnings, savings, assets,*
ii. *Subsistence farming*
iii. *Social security (e.g. old-age pension)*
iv. *Wealth transfers from family or friends (if family, who?)*
v. *Other income source*

F. Old age and support

17. Do you need help during the day?

a. Think about the past week or month, did you need support for anything from others?
b. Are you dependent on support from others?

18. For what do you need help?

a. Think about the past week or month, what were typical situations in which you needed support?
b. In which respect are you dependent on support from others?

19. Who is helping you when you need help?

a. To whom do you go to if you need help?
b. Who will be taking care of you in the future?
c. Are you worried about who will be taking care of you?

Following, I would like to concentrate on your adult children (talking about your children between 18 and 59 years old).

G. Offspring and support

20. Could you please describe your relationship to your adult child/children?

a. How often do you see your child/children?
b. How often do you interact with them?
c. How much time do you spend with your child/children?
d. How do you spend your time with your child/children?

Possible answers:

i. *Personally, phone-call, other possible circumstances*

21. Is/Are your adult child/children supporting you?

a. If not, would you like your child/children to support you?
b. In which way would you like your child/children to help you?

22. How is/are your adult child/children currently supporting you?

a. Providing money
b. Providing household assets (e.g., fridge, car) or utilities (e.g., electricity)
c. Help with economic activities (e.g., agricultural work, selling goods)
d. Help with fetching water for the house
e. Help with herding animals or taking care of livestock
f. Help with other domestic activities (e.g., shopping, cooking, cleaning)
g. Help with languages (e.g., English translation)
h. Help navigating health services, social services, or old-age pension

i. Providing you with other knowledge or any skills<br>j. Other

HIV and HIV-treatment have been a topic in your family. I would like to encourage you to tell me as much as you want to about HIV among your adult children. Please keep in mind that anything you say will be kept confidential.

**H. Offspring and HIV**
**23. What do you know about the HIV infection among your adult child/children?**<br>a. Can you tell from your experience about HIV among your child/children?
**24. Among your children, do you know how many of them are tested for HIV?**<br>a. How many of them are tested HIV positive? *(If no HIV test positive exclusion criteria!)*<br>b. Among your children tested HIV positive, do you know how many of them are treated with HIV treatment?<br>c. Among your children tested HIV positive, do you know how many of them are virally suppressed?
**25. Please walk me through the process of you children getting tested HIV-positive**<br>a. How did you and your child/children go through the process of having to deal with HIV?<br>b. What did you think when your child/children got the diagnosis to be HIV-positive?
**26. Did the HIV diagnosis among your child/children have consequences for you?**<br>*a. Positive consequences?*<br>*b. Negative consequences?*<br>c. How has your life changed since the HIV-infection of your *SON/DAUGHTER to now*?<br>*Possible domains:*<br>*i. Health (physical and psychological)*<br>*ii. Work*<br>*iii. Economic situation*<br>*iv. Caring for grandchildren*<br>*v. Status/Reputation (Stigmatization/Discrimination)*<br>*vi. Social environment*<br>*vii. Other effects (please specify)*
**27. How does/did HIV among your adult child/children affect the support you receive?**<br>a. How did the infection/disease affect your support structures?
**28. How does/did HIV among your adult child/children affect your family?**<br>a. How does the diagnosis affect the relationship within your family?<br>b. How does the diagnosis change household structures?
**29. How does your family deal with the topic of HIV/AIDS among your adult child/children?**<br>a. How do you deal with the topic of HIV/AIDS in your family?<br>b. Is HIV/AIDS an openly discussed topic in your family?<br>c. Does everybody in the family know about the HIV-infection among your adult children?

Are you familiar with the term 'Antiretroviral therapy' in short 'ART'? This term describes the HIV-treatment. For the following questions I would like to ask you about HIV-treatment among your adult child/children. *[Use term for 'ART' that respondent is familiar with!]*

**I. Working-age and ART**
**30. Do you remember the first time you heard about Antiretroviral therapy/HIV-treatment?**<br>a. When was the first time you heard of ART?

31. **Has the HIV-treatment been a topic openly discussed in your family?**

32. **What do you know about HIV treatment in your family?**
    a. What do you know about HIV-treatment among your HIV positive adult child/children?
    b. Are you informed about the treatment status of your child/children?
    c. Do you ask him/her/them regularly about their treatment with Antiretroviral therapy?
    d. Has ART been consistently available to your co-residing HIV positive offspring?

33. **Please describe from your point of view how HIV-treatment has changed the life of your <u>adult HIV-positive child/children</u>.**
    a. Are there positive changes?
    b. Are there negative changes?
    c. Is there anything that your child/children could not do before receiving ART?
    d. Is there anything that your child/children started doing after receiving ART?
    e. Has ART affected your adult children's life plans?
    f. Has ART affected your adult children's ability to go back to work?
    g. Do you think that ART affects your adult children's life expectancy?
    h. Do you think that ART improves the reputation of your child?

J. Offspring and ART

34. **Has HIV-treatment among your adult child/children affected <u>you</u>?**
    a. How has your life changed since HIV treatment of your *SON/DAUGHTER to now*?
    b. Are there any consequences of HIV treatment among your adult child/children for you?
        i. Positive effects?
        ii. Negative effects?
    c. Did HIV treatment affect:
        i. Your adult children's ability to send/give you money?
        ii. Providing household assets (e.g., fridge, car) or utilities (e.g., electricity)
        iii. Your reputation/discrimination/stigmatization (house, village, community)
        iv. Help with economic activities (e.g., agricultural work, selling goods)
        v. Help with fetching water for the house
        vi. Help with herding animals or taking care of livestock
        vii. Help with other domestic activities (e.g., shopping, cooking, cleaning)
        viii. Your adult children's ability to provide direct care to you?
        ix. Your ability to access the health system (e.g., health center), social services, or grants (e.g., old-age pension)?
        x. Your health knowledge or knowledge of the health system?
        xi. Effects on your perceptions of "modern medicine" (vis-a-vis traditional healers)?
        xii. Effects on your health-seeking behavior (clinic vs. traditional healer)?
        xiii. Other?

35. **Do you feel that the overall consequences of ART among your adult child/children are positive or negative for you?**
    b. …for your status? If so,why?
    c. …for your family? If so,why?
    d. …for your social environment? If so,why?
    e. …for your broader community? If so,why?

36. **Do you think that further testing for HIV in your community would be good?**

37. **Would further expanding access to HIV treatment in your community have benefits or disadvantages for the family?**
    a. If so, please explain.

I will now give you a piece of paper and a pencil. Please make a mark on the line according to the questions that I will ask you. Please share your thoughts with me while working on the questions.

K. Timeline of support
**38. Present**<br>a. How much support do you receive from your adult child/children today?
**39. Past**<br>a. How much support did you receive from your adult child/children in the past (before HIV diagnosis of child/children)?
**40. HIV diagnosis**<br>a. How much support did you receive from your adult child/children at the time of his/her HIV diagnosis?
**41. HIV treatment**<br>a. How much support did you receive from your adult child/children at the time he/she started taking HIV treatment?
**42. Other event**<br>a. Is there an event that changed the way your adult child/children did support you?<br>b. How much support did you receive from your adult child/children at the time of this event?

L. HIV and ART and stigma/disclosure
**43. Do you think people keep the HIV diagnosis a secret?**<br>If so, why do you think people keep HIV a secret and do not talk about it?
**44. Do you think that HIV treatment can improve the stigma of HIV and AIDS?**
**45. Do you think that men and women do things differently with respect to HIV and treatment?**<br>If so, what do you think they are doing differently?<br>b. Do they differ in:<br>i. HIV-testing?<br>ii. Health seeking behaviour (going to clinic)?<br>iii. Disclosing the status?<br>iv. Talking freely about related topic?<br>v. Adhering to HIV treatment?<br>vi. Talking open about HIV treatmen?

M. Conclusion
**46. Is there a question that you think of important and which I have not asked during this conversation?**<br>b. Is there anything else you would like to share that hasn't been voiced?

Before we come to an end, I would like to check a list with you to see if I have asked you all important questions. *[Only complete what has not been covered, otherwise skip section]*

N. Quantitative data
**47. Respondent's:**<br>a. Personal data<br>i. Age<br>ii. Gender<br>iii. Marital status/Partnership

iv. Schooling level (as either e.g., no formal schooling; some primary; primary completed; some secondary; or secondary schooling or higher)

f. Family
    i. Number of children
    ii. Age of children
    iii. Children's place of residence
    iv. Age of grandchildren
    v. Number of grandchildren
    vi. Biological/social parents alive/dead
    vii. Age of parents

g. Home and living circumstances
    i. Place of residence
    ii. Home (How does respondent live? House, apartment, single room, shared living-space, possible other living circumstances)
    iii. Head of household
    iv. Exact number of household members
        1. Partner
        2. Parents
        3. Children
        4. Grandchildren
        5. Friends
        6. Others *(please specify)*

h. Work life/Retirement status
    i. Current occupation
    ii. Work status/retirement status
    iii. Economic support structures
    iv. Economic situation: Earnings, savings, assets
    v. Social security (e.g. old-age pension)

i. HIV status and HIV treatment
    i. Date of HIV diagnosis
    ii. Date of start of HIV treatment
    iii. HIV treatment status (Not virally suppressed, virally suppressed)
    iv. Date of start with ART
    v. Compliance to ART
        1. Attending appointments regularly?
        2. Taking the HIV treatment regularly?
    vi. Number of appointments at health clinics
    vii. HIV-related symptoms
    viii. ART-related symptoms

Thank you very much for sharing all your answers with me. We are at the end of the interview. I will stop the recording.

[Stop recording]

Do you have questions concerning the interview or the study?

Thank you for your participation!

File S3. Participant sheet: timeline of support young or middle-aged respondent (English)

Timeline of Support

Working-age respondent

1. **Present**
   How much **do** you support your older family member(s) **today**?

From **'No support'** to **'Full support'.** Please mark on the line.

No support |—|—|—|—|—|—|—|—|—|—| **Full support**

2. **Past**
   How much **did** you support your older family member(s) **in the past before your HIV diagnosis**?

From **'No support'** to **'Full support'.** Please mark on the line.

No support |—|—|—|—|—|—|—|—|—|—| **Full support**

3. **HIV diagnosis**
   How much **did** you support your older family member(s) **at the time of your HIV diagnosis**?

From **'No support'** to **'Full support'.** Please mark on the line.

No support |—|—|—|—|—|—|—|—|—|—| **Full support**

4. **HIV treatment**
   How much **did** you support your older family member(s) **at the time you started taking HIV treatment**?

From **'No support'** to **'Full support'.** Please mark on the line.

No support ├─┼─┼─┼─┼─┼─┼─┼─┼─┼─┤ **Full support**

5. **Other event** Is there an event that changed the way you supported your older family member(s)? Please name event and year:..

   How much **did** you support your older family member(s) **at the time of this event**?

From **'No support'** to **'Full support'.** Please mark on the line.

No support ├─┼─┼─┼─┼─┼─┼─┼─┼─┼─┤ **Full support**

File S4. Participant sheet: timeline of support older respondent (English)

Timeline of Support

Older respondent

1. **Present**
   How much support **do** you receive from your adult child/children **today**?

From **'No support'** to **'Full support'.** Please mark on the line.

No support ├──┼──┼──┼──┼──╋──┼──┼──┼──┼──┤ **Full support**

2. **Past**
   How much support **did** you receive from your adult child/children in the **past before HIV diagnosis of child/children**?

From **'No support'** to **'Full support'.** Please mark on the line.

No support ├──┼──┼──┼──┼──╋──┼──┼──┼──┼──┤ **Full support**

3. **HIV diagnosis** (HIV diagnosis of adult child/children)
   How much support **did** you receive from your adult child/children **at the time of his/her HIV diagnosis**?

From **'No support'** to **'Full support'.** Please mark on the line.

No support ├──┼──┼──┼──┼──╋──┼──┼──┼──┼──┤ **Full support**

4. **HIV t**[illegible] ildren)
   How much support **did** you receive from your adult

child/children **at the time he/she started taking HIV treatment**?

From **'No support'** to **'Full support'.** Please mark on the line.

No support |—|—|—|—|—|—|—|—|—|—| **Full support**

5. **Other event** Is there an event that changed the way your adult child/children **did** support you? Please name year and event: .... How much support **did** you receive from your adult child/children **at the time of this event**?

From **'No support'** to **'Full support'.** Please mark on the line.

No support |—|—|—|—|—|—|—|—|—|—| **Full support**

References for Online Supplementary Document

1. South African Government. Old age pension. 2023. https://www.gov.za/services/social-benefits-retirement-and-old-age/old-age-pension (accessed 10 May 2023).

2. Kimuna SR, Makiwane M. Older people as resources in South Africa: Mpumalanga households. *J Aging Soc Policy* 2007; **19**(1): 97-114.

3. Ralston M, Schatz E, Menken J, Gómez-Olivé FX, Tollman S. Who Benefits--Or Does not--From South Africa's Old Age Pension? Evidence from Characteristics of Rural Pensioners and Non-Pensioners. *Int J Environ Res Public Health* 2015; **13**(1): 85.

4. Case A, Menendez A. Does money empower the elderly? Evidence from the Agincourt demographic surveillance site, South Africa. *Scand J Public Health Suppl* 2007; **69**: 157-64.

5. Schatz E, Gómez-Olivé X, Ralston M, Menken J, Tollman S. The impact of pensions on health and wellbeing in rural South Africa: does gender matter? *Soc Sci Med* 2012; **75**(10): 1864-73.
